# Whole genome analysis of local Kenyan and global sequences unravels the epidemiological and molecular evolutionary dynamics of RSV genotype ON1 strains

**DOI:** 10.1093/ve/vey027

**Published:** 2018-09-24

**Authors:** J R Otieno, E M Kamau, J W Oketch, J M Ngoi, A M Gichuki, Š Binter, G P Otieno, M Ngama, C N Agoti, P A Cane, P Kellam, M Cotten, P Lemey, D J Nokes

**Affiliations:** 1 Epidemiology and Demography Department, Kenya Medical Research Institute (KEMRI) – Wellcome Trust Research Programme, P.O. Box 230, 80108 Kilifi, Kenya; 2 Virus Genomics, Wellcome Trust Sanger Institute, Hinxton, Cambridge,UK; 3 Kymab Ltd., Babraham Research Campus, Cambridge, UK; 4 Department of Biomedical Sciences, Pwani University, Kilifi, Kenya; 5 High Containment Microbiology, Public Health England, Salisbury, UK; 6 Division of Infectious Diseases, Department of Medicine, Imperial College London, London, UK; 7 Department of Viroscience, Erasmus Medical Center, Rotterdam, The Netherlands; 8 Department of Microbiology and Immunology, KU Leuven – University of Leuven, Leuven, Belgium; 9 School of Life Sciences and Zeeman Institute for Systems Biology and Infectious Disease Epidemiology Research (SBIDER), University of Warwick, Coventry, UK

**Keywords:** virus evolution, respiratory syncytial virus, RSV, ON1, genomic epidemiology, phylodynamics

## Abstract

The respiratory syncytial virus (RSV) group A variant with the 72-nucleotide duplication in the G gene, genotype ON1, was first detected in Kilifi in 2012 and has almost completely replaced circulating genotype GA2 strains. This replacement suggests some fitness advantage of ON1 over the GA2 viruses in Kilifi, and might be accompanied by important genomic substitutions in ON1 viruses. Close observation of such a new virus genotype introduction over time provides an opportunity to better understand the transmission and evolutionary dynamics of the pathogen. We have generated and analysed 184 RSV-A whole-genome sequences (WGSs) from Kilifi (Kenya) collected between 2011 and 2016, the first ON1 genomes from Africa and the largest collection globally from a single location. Phylogenetic analysis indicates that RSV-A circulation in this coastal Kenya location is characterized by multiple introductions of viral lineages from diverse origins but with varied success in local transmission. We identified signature amino acid substitutions between ON1 and GA2 viruses’ surface proteins (G and F), polymerase (L), and matrix M2-1 proteins, some of which were positively selected, and thereby provide an enhanced picture of RSV-A diversity. Furthermore, five of the eleven RSV open reading frames (ORFs) (G, F, L, N, and P) formed distinct phylogenetic clusters for the two genotypes. This might suggest that coding regions outside of the most frequently studied G ORF also play a role in the adaptation of RSV to host populations, with the alternative possibility that some of the substitutions are neutral and provide no selective advantage. Our analysis provides insight into the epidemiological processes that define RSV spread, highlights the genetic substitutions that characterize emerging strains, and demonstrates the utility of large-scale WGS in molecular epidemiological studies.

## 1. Introduction

Respiratory syncytial virus (RSV) is the leading viral cause of severe pneumonia and bronchiolitis among infants and children globally (Nokes et al. 2008; Nair et al. 2010; Shi et al. 2017). Individuals remain susceptible to RSV upper respiratory tract reinfection throughout life even though they develop immune responses following primary and secondary RSV infections in childhood (Agoti et al. 2012). No licensed RSV vaccine exists, partly due to the antigenic variability in the virus (Cane 2001).

The single stranded, negative sense RSV genome encodes eleven proteins of which the attachment glycoprotein (G) is the most variable and a key player of adaptive evolution of the virus (Cane and Pringle 1995). RSV is classified into two groups, RSV-A and RSV-B (Mufson et al. 1985), differing antigenically (Sande et al. 2013), with each group further characterized into genotypes [with genotype defined as a cluster of viruses each of which has greater genetic distance from viruses of any other genotype compared to that between viruses of the most diverse genotype (Peret et al. 1998; Trento et al. 2015)]. A genotype can be further divided into (1) imported variants which show greater genetic difference than expected from *in situ* diversification (Agoti et al. 2015b; Otieno et al. 2016) and (2) local variants arising from recent introduction which subsequently diversify *in situ* (without time for purifying selection from, for example inter-epidemic bottlenecks) (Agoti et al. 2017). We have previously shown that within RSV epidemics, there is co-circulation of RSV viruses belonging to different groups, genotypes, and variants both imported and local (Agoti et al. 2015b, 2017; Otieno et al. 2016), with the latter not clearly distinguished through partial G gene sequencing. Consequently, full genome sequencing offers the opportunity to differentiate introduced from persistent RSV viruses within a given location.

Two recent RSV genotypes with large duplications within the G glycoprotein, BA and ON1, have been detected globally. The RSV-B BA genotype is characterized by a 60-nucleotide (nt) duplication while the RSV-A ON1 genotype by a 72-nucleotide duplication. Initially detected in Buenos Aires Argentina in 1999, the BA genotype spread rapidly throughout the world becoming the predominant group B genotype and replacing all previous circulating RSV-B genotypes in certain regions (Trento 2003; Trento et al. 2010). The ON1 genotype was first detected in 2010 in Ontario Canada, a decade after BA, and has also spread globally (Eshaghi et al. 2012; Prifert et al. 2013; Tsukagoshi et al. 2013; Valley-Omar et al. 2013; Agoti et al. 2014; Auksornkitti et al. 2014; Pierangeli et al. 2014; Avadhanula et al. 2015; Duvvuri et al. 2015). Of interest is what could be driving the apparent fitness advantage of these emergent genotypes over the preceding genotypes (Hotard et al. 2015), and whether such insights could be mined from whole-genome sequences (WGSs).

The rate of nucleotide substitution for the G gene encoding the attachment protein has been estimated to be 1.83 × 10^−3^ and 1.95 × 10^−3^ nucleotide substitutions/site/year for groups A and B, respectively, with some variation dependent on the timescale of observation (Zlateva et al. 2004, 2005). Similarly, although at a lower rate, there is also significant ongoing accumulation of substitutions across the rest of the genome (Agoti et al. 2015a, 2017). At present, there is limited knowledge about the selective forces acting on genes other than the G gene as a result of paucity of WGSs, particularly from the same location over a period spanning multiple seasons (Tan et al. 2012, 2013). Therefore, genetic signatures across the rest of the genome that might additionally inform on the adaptive mechanisms of RSV viruses following introduction into communities have not been investigated before.

In this study, we sought to gain a deeper understanding of the epidemiological and evolutionary dynamics of RSV viral populations through extensive whole-genome sequencing and analysis of samples collected as part of on-going surveillance studies of respiratory viruses within Kilifi, Coastal Kenya (2011–16). This WGS analysis advances previous work on the patterns of introduction and persistence of the ON1 variant within this community that utilized partial G gene sequences (Agoti, Otieno, and Gitahi 2014; Otieno et al. 2017), and provides a higher resolution of the RSV genetic structure, spread and identification of variation that may be associated with molecular adaptation and apparent fitness advantages.

## 2. Materials and methods

### 2.1 Ethics statement

The samples obtained in Kilifi were collected following informed written consent from each child’s guardian or parent. KEMRI Ethical Review Board, Kenya, and the Coventry Research Ethics Committee of the UK approved the study protocols (Nokes et al. 2008, 2009).

### 2.2 Study population

This study is part of ongoing surveillance of respiratory viruses within Kilifi County, coastal Kenya, and across the country that is aimed at understanding the epidemiology and disease burden of respiratory viruses in this region (Nokes et al. 2009). Two sets of samples were used in the current analysis: (1) samples collected from children (under 5 years of age) admitted to the Kilifi County Hospital (KCH) presenting with syndromically defined severe or very severe pneumonia between September 2011 and August 2016 (Nokes et al. 2009; Otieno et al. 2017) and (2) samples collected from patients of all ages presenting at health facilities within the Kilifi Health and Demographic Surveillance System (KHDSS) (Scott et al. 2012) with acute respiratory illnesses between January and December 2016 (Nyiro et al. 2018).

### 2.3 RNA extraction and PCR amplification

**Figure 1.**
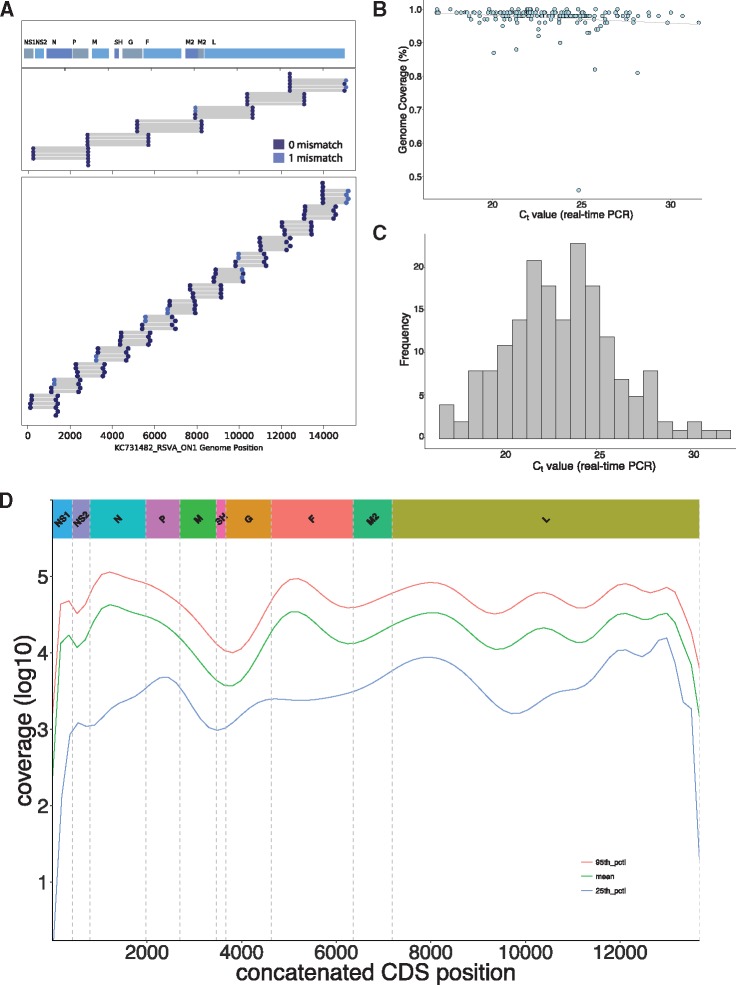
Sample sequencing and genome details. The two RSV-A whole genome amplification strategies used in this study are shown in (A), i.e. six and fourteen amplicons. For each panel the positions of primer targets for each amplicon are indicated. The locations of the eleven RSV ORFs are indicated on top of panel 1. (B) The proportion of RSV genome length sequence recovered (using KC731482 as the reference) for all the 184 genomes was plotted as a function of sample’s diagnostic real-time PCR Ct value. (C) The distribution of the diagnostic real-time PCR Ct values for the 184 sequenced samples reported here (KCH and KHDSS). (D) The log values of the sequencing depth (see Materials and methods) at each position of the genome assemblies along the concatenated RSV ORFs (i.e. excluding the intergenic regions).

All KCH admissions specimens had previously been screened for RSV (immunofluorescent antibody test, IFAT), RSV group (multiplex real-time polymerase chain reaction) and RSV-A genotype status (G gene amplification followed by Sanger sequencing), and partial G-gene sequencing results reported (Otieno et al. 2017), while the KHDSS samples were screened afresh using the same multiplex real-time PCR methods referred to as above. To pick samples proceeding to WGS, we selected (1) all the RSV-A positives from the KHDSS, (2) all the GA2 positives from KCH, and (3) a random subsample (50%) of the ON1 positives per epidemic from the KCH. Additionally, we targeted samples with real-time PCR cycle threshold (Ct) value < 30 based on the success rate from previous experience (Agoti et al. 2015a), with the exception of four test samples that were PCR negative or had Ct > 30. Viral RNA was extracted using QIAamp Viral RNA Mini Kit (QIAGEN, London, UK). Reverse transcription of RNA molecules and PCR amplification were performed with a six-amplicon, six-reaction strategy (Agoti et al. 2015a), or using a six or fourteen-amplicon strategy (unpublished) split into two reactions of three and seven amplicons, respectively for each, Fig. 1A. Amplification success was confirmed by observing the expected PCR product size (1200–1500 bp) on 0.6% agarose gels. Amplicons from six or two reactions were pooled and purified for Illumina library preparation.

### 2.4 Illumina library construction and sequencing

The purified PCR products were quantified using Qubit fluorimeter 2.0 (Life Technologies) and normalized to 0.2 ng/μl. The normalized DNA was tagmented (a process of fragmentation and tagging) using the Nextera XT (Illumina, San Diego, CA, USA) library prep kit as per the manufacturer’s instructions. Indices were ligated to the tagmented DNA using the Nextera XT index kit (Illumina). The barcoded libraries were then purified using 0.65X Ampure Xp beads. Library quality control was carried out using the Agilent high sensitivity DNA kit on the Agilent 2100 Bioanalyzer (Agilent, Waldbronn, Germany) to confirm the expected size distributions and library quality. Each library was quantified using the Qubit fluorimeter 2.0 (Life Technologies), after which the libraries were normalized and pooled at equimolar concentrations. The pooled libraries were sequenced on either (1) Illumina HiSeq system using 2 × 250 bp paired-end (PE) sequencing at the Wellcome Trust Sanger Institute (UK) or (2) Illumina MiSeq using 2 × 250 bp PE sequencing at the KEMRI-Wellcome Trust Research Programme (Kilifi, Kenya).

A preliminary quality check of the sequence reads was done using fastqc (Andrews 2010) with the output per batch aggregated and visualized by multiqc (Ewels et al. 2016). To determine the proportion of RSV and non-RSV reads in the samples used here, Kraken v0.10.6 (Wood and Salzberg 2014) was used with a pre-built Kraken database provided by the viral-ngs pipeline (Park et al. 2015; Park, Tomkins-Tinch, and Ye 2016) (downloaded in December 2015; https://storage.googleapis.com/sabeti-public/meta_dbs/ kraken_ercc_db_20160718 .tar.gz).

### 2.5 Depletion of human reads

Prior to deposition of the raw short reads into NCBI short read archive, datasets were depleted of human reads. The raw reads were mapped onto the human reference genome hg19 using bowtie2 (Langmead et al. 2009) while samtools (Li et al. 2009) was used to filter, sort, and recover the unmapped (nonhuman) reads. The final reads are available in the NCBI BioProject database under the study accession PRJNA438443.

### 2.6 Genome assembly and coverage

Consensus genome assemblies were generated either using viral-ngs versions 1.18.0/1.19.0 (Park et al. 2015; Park, Tomkins-Tinch, and Ye 2016) and/or SPAdes version 3.10.1 (Bankevich et al. 2012), selecting the most complete assembly from either assemblers. The available Sanger G-gene sequences (Agoti, Otieno, and Gitahi 2014; Otieno et al. 2017) for these samples were additionally used to confirm agreement with the WGS assemblies. The genomes generated in this study are available in GenBank under accession numbers MH181878–MH182061. The genomes were aligned using MAFFT alignment software v7.305 (Katoh and Standley 2013) using the parameters ‘–localpair –maxiterate *1000*’.

To calculate and visualize depth of coverage, sample raw reads were mapped onto individual assemblies with BWA (Li and Durbin 2009), samtools (Li et al. 2009) were used to sort and index the aligned bam files, and finally bedtools (Quinlan and Hall 2010) were used to generate the coverage depth statistics. Plotting of the depth of coverage was done in R (R Core Team 2015) in the RStudio (RStudio Team 2016).

### 2.7 Global comparison dataset

All complete and partial genome sequences available in GenBank Nucleotide database (https://www.ncbi.nlm.nih.gov/genbank/) as on 19 September 2017 were used to prepare a global RSV-A genotype ON1 genomic and G-gene dataset. To prepare the global ON1 dataset, we downloaded all RSV sequences from GenBank (search terms: respiratory syncytial virus), created a local blast database in Geneious (Kearse et al. 2012), and performed a local blast search using the 144 nucleotide sequence region of the ON1 genotype. To remove duplicates, the sequences were binned by country of sample collection, filtered of duplicates and then re-collated into a single dataset. For the global G-gene dataset of 1,167 sequences, the sequence length ranged from 238 to 690 bp. The final alignment of 344 ON1 genome sequences comprised the sequences reported in this study (*n *=* *154) and additional publicly available GenBank ON1 sequences (*n *=* *190). In addition to the ON1 genomes, we generated thirty genotype GA2 genome sequences from Kilifi. The alignments were inspected in AliView (Larsson 2014) and edited manually removing unexpected spurious frame-shift indels (largely homopolymeric and most likely sequencing errors).

### 2.8 Maximum likelihood phylogenetic analyses and root-to-tip regression

Separate Maximum-Likelihood (ML) phylogenetic trees were generated using multiple sequence alignments of the three datasets, i.e. Kilifi WGS, and global G-gene and WGS datasets. The ML trees were inferred using both PhyML and RaxML, with each optimizing various parts of the tree generation process (i.e. borrowing strengths of both approaches), using the script generated and deposited by Andrew Rambaut at (https://github.com/ebov/space-time/tree/master/Data/phyml_raxml_ML.sh). The GTR+G model was used after determination as the best substitution model by IQ-TREE v.1.4.2 (Chernomor, von Haeseler, and Minh 2016).

To determine presence of temporal signal (‘clockiness’) in our datasets, we used TempEst v1.5 (Rambaut et al. 2016) to explore the relationship between root-to-tip divergence and sample dates. The data were exported to R (R Core Team 2015) to perform a regression with the ‘lm’ function.

### 2.9 Estimating the number of local variant introductions

To differentiate between local variants arising from a recent introduction and imported variants with greater genetic differences than is expected from local diversification, we used a pragmatic criterion previously described by Agoti et al. (2015b). Briefly, a variant is a virus (or a group of viruses) within a genotype that possesses ≥*x* nucleotide differences compared to other viruses. This *x* nucleotide differences is a product of the length of the genomic region analysed, estimated substitution rate for that region, and time. This analysis was done using usearch v8.1.1861 (Edgar 2010).

### 2.10 Protein substitution and selection analysis

Using the aligned Kilifi (ON1 and GA2) genome dataset, patterns of change in nucleotides (single nucleotide polymorphisms or SNPs) and amino acids were sought using Geneious v11.1.2 (Kearse et al. 2012) and BioEdit 7.2.5 (Hall 1999), respectively. Potential positively selected and co-evolving sites within the coding regions were identified using HyPhy (Pond, Frost, and Muse 2005) and phyphy (Spielman 2018). SNPs were called from both the complete dataset and from an alignment of the consensus sequences from GA2 and ON1, whereby a consensus nucleotide was determined as the majority base at a given position. For the positive selection analysis, two strategies were used; gene-wide selection detection [BUSTED (Murrell et al. 2015)] and site-specific selection [SLAC, FEL (Kosakovsky Pond et al. 2005), FUBAR (Murrell et al. 2013), and MEME (Murrell et al. 2012)]. Codon positions with a *P*-value <0.1 for either the SLAC, FEL, and MEME models or with a posterior of probability >0.9 for the FUBAR method were considered to be under positive selection.

### 2.11 Bayesian phylogenetics

To infer time-structured phylogenies, Bayesian phylogenetic analyses were performed using BEAST v.1.8.4 (Drummond et al. 2012). Because of sparse data at the 5′ and 3′ termini and in the noncoding regions of the genomic datasets, only the coding sequences (CDSs) were used as input. The SRD06 substitution model (Shapiro, Rambaut, and Drummond 2006) was used on the CDS and three coalescent tree priors were tested, i.e. a constant-size population, an exponential growth population, and a Bayesian Skyline (Drummond et al. 2005). For each of these tree priors, combinations with the strict clock model and an uncorrelated relaxed clock model with log-normal distribution (Drummond et al. 2006) were tested with the molecular clock rate set to use a noninformative continuous time Markov chain rate reference prior (Ferreira and Suchard 2008). For each of the molecular clock and coalescent model combinations, the analyses were run for 150 million Markov Chain Monte Carlo (MCMC) steps and performed both path-sampling and stepping-stone to estimate marginal likelihood (Baele et al. 2012, 2013). The best fitting model was a relaxed clock with a Skyline coalescent model, Supplementary Table S1.

BEAST was then run with 300–400 million MCMC steps using the SRD06 substitution model, Skyline tree prior, and relaxed clock model to estimate Bayesian phylogenies. For the time to the most recent common ancestor (TMRCA) estimates, the same substitution model and tree prior were used as above but with a strict clock model. For the global G-gene dataset, BEAST was run with 400 million MCMC steps using the HKY substitution model, Skyline tree prior, and a relaxed clock model. We used Tracer v1.6 to check for convergence of MCMC chains and to summarize substitution rates. Maximum clade credibility (MCC) trees were identified using TreeAnnotator v1.8.4 after removal of 10% burn-in and then visualized in FigTree v1.4.3.

### 2.12 Principal component analysis

To check on any clustering and stratification patterns, principal component analysis (PCA) was performed using the R package FactoMineR (Lê, Josse, and Husson 2008). The input data were a matrix of pairwise distances from genome sequence alignment using the ‘N’ model of DNA evolution, i.e. the proportion or the number of sites that differ between each pair of sequences. Each genome on the PCA plot was annotated by the continent of sample origin.

## 3. Results

### 3.1 Genome sequencing and assemblies

Over the five RSV epidemics sampled (2011/2012 to 2015/2016), a total of 3,157 samples were collected from eligible children at KCH, 3,146 (99.7%) were tested for RSV by IFAT or real-time PCR, and 801 (25.5%) RSV positives identified. Of these, 434 (54.2%) were RSV-A, of which 412 (94.9%) were successfully sequenced from routine G-gene sequencing, with 354 (85.9%) of genotype ON1 and the remainder 58 of genotype GA2. From the peripheral health centres within the KHDSS, a total of thirty-two RSV-A positives were identified by real-time PCR.

A total of 184 RSV-A genomes were generated in this study, comprising genotypes ON1 (*n *=* *154) and GA2 (*n *=* *30); Supplementary Table S2. This dataset included 176 genomes from inpatients at KCH and 8 genomes from the KHDSS. The sequencing success for KCH samples was 87% (154 full genomes/177 samples processed for sequencing) for ON1 viruses (the denominator a 50% sub-sample of all 354) and 52% (30 full genomes/58 samples processed for sequencing) for GA2 viruses, and for KHDSS samples was 25% (8 full genomes/32 samples processed for sequencing). The Ct values for KHDSS samples (as an indicator of viral load) had a median of 26.3 [Interquartile Range (IQR): 22.9–28.0], which was slightly higher than for the KCH sample set with a median Ct of 24.4 (IQR: 22.2–26.9), Supplementary Fig. S2. Between 0.2 and 4.3 million short reads were available per sample of which RSV-specific reads ranged between 0.001 and 3.9 million reads. The genome assemblies had a median length of 15,054 nucleotides (range: 13,966–15,322) and mean depth of base coverage per genome ranging from 39 to 66,457.

Whereas the samples for WGS were generally of high viral content (lower Ct value), it is apparent there was reduced genome yield (proportion of genome assembled) from samples with lower viral loads (i.e. higher Ct values); Fig. 1B. However, the samples successfully sequenced and analysed here generally had lower Ct values (higher viral loads) as shown in Fig. 1C. The median fraction of the genome with unambiguous base calls was 98% with reference length from KC731482. Read coverage across the genomes was nonuniform, Fig. 1D, suggesting varied PCR amplification efficiency among primer pair combinations combined with increased sequencing yield from the ends of the amplicons.

### 3.2 Bayesian reconstruction of ON1 epidemiological and evolutionary history

**Figure 2.**
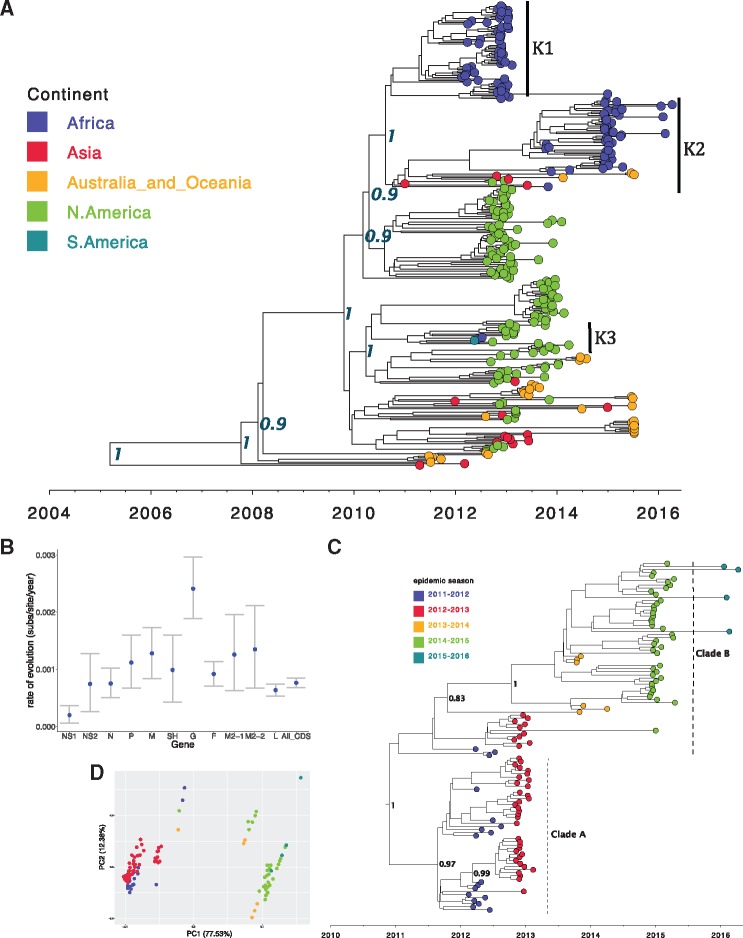
Global and local ON1 MCC trees and PCA. (A) MCC tree inferred from 344 global full genome sequences (see Materials and methods) with the tips colour coded with the continent of sample collection. All the African samples (in blue, K1-3 and vertical bars) in this dataset were only available from Kilifi (Kenya). Node labels are posterior probabilities indicating support for the selected nodes. (B) The evolutionary rate estimates for the different genotype ON1 ORFs. (C) An MCC tree inferred from 154 ON1 genomes from Kilifi annotated with identified lineages A and B, and the tips colour coded with the epidemic season. (D) A PCA analysis (see Materials and methods) of the same dataset as (C) and similarly annotated with the epidemic season. Percentage of variance explained by each component is indicated on the axis.

The global ON1 whole-genome MCC phylogenetic tree, Fig. 2A, shows evolutionary relationship among ON1 viruses from five sampled continents. The TMRCA of the ON1 strains from the most recent tip (7 April 2016) was estimated to be 11.07 years [95% HPD: 9.85–12.31], resulting in an estimated ON1 emergence date of between December 2003 and June 2006. This estimated date of emergence is earlier than a previous estimate (2008–09) using the G-gene alone (Duvvuri et al. 2015), but such a difference could be a reflection of the different datasets (by geography and sampling dates). Comas-Garcia et al. have reported the earliest ON1 strain identified to date in November 2009 from central Mexico (Comas-García et al. 2018), and from our estimates this suggests a period of 3–6 years of circulation of this virus before first detection. The genome-wide substitution rate for the ON1 viruses was estimated at 5.97 × 10^−4^ nucleotide substitutions per site per year [95% HPD: 5.42–6.58 × 10^−4^], similar to previous estimates for RSV group A full length sequences sampled over several epidemics (Tan et al. 2013; Agoti et al. 2015a) but slower than estimates from samples collected from a household study over a single epidemic within the same location and from using a global ON1 G-gene dataset (Duvvuri et al. 2015; Agoti et al. 2017). Across the genome, estimates of evolutionary rates for individual ON1 open reading frames (ORFs) varied, Fig. 2B, with the mean substitution rate highest in the G-gene, lowest in NS1, and moderate (with tight 95% HPD intervals) for the whole genome.

The Kilifi ON1 genomes were placed at three lineages (K1-3 and black vertical bars) on the global tree in Fig. 2A. However, when the Kilifi ON1 WGS were analysed separately, Fig. 2C, two lineages were observed (labelled A and B) with a temporal grouping whereby A comprised sequences from the 2011–13 RSV epidemic period while B comprised sequences predominantly from the epidemic period 2013–16. These lineages and temporal patterns are further highlighted by the PCA analysis in Fig. 2D. Based on the phylogenetic placement of the Kilifi ON1 genomes on the global tree in Fig. 2A, we estimate that there could have been at least three separate introductions of ON1 viruses into Kilifi. One of these potential Kilifi ON1 introductions (K3) was characterized by only two cases, which is consistent with limited local transmission. In addition, the eight outpatient ON1 viruses collected from the KHDSS were interspersed with viruses sampled from inpatient admissions at KCH suggesting that our sampling at the hospital might be well representative of the larger KHDSS community.

Using the global whole genome ON1 substitution rate estimate above, the Kilifi ON1 genomes dataset (length 15,404 bp) and a pragmatic criterion previously described by Agoti et al. (2015b) to differentiate between local and imported variants, we estimated that there were up to 73 ON1 introductions into Kilifi. Even when we used the higher substitution rate previously estimated from ON1 partial G-gene sequences by Duvvuri et al. (2015), i.e. 4.10 × 10^−3^ substitutions/site/year which translates to a difference of at least sixty-three nucleotides between any two genomes to be classified as separate introductions, this resulted in an estimate of six separate introductions. This suggests that multiple seeding introductions of ON1 viruses may have been required to sustain their local transmission.

### 3.3 Global ON1 spatiotemporal dynamics

**Figure 3.**
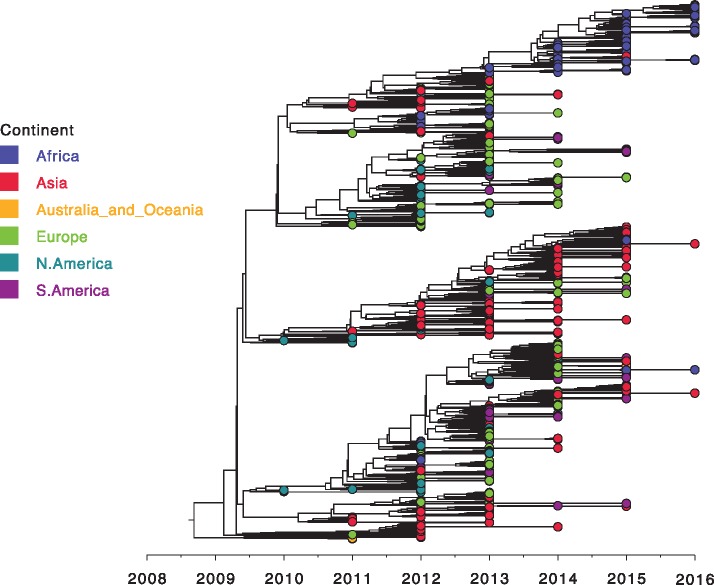
Global ON1 G-gene MCC phylogenetic tree. An MCC tree inferred from 1,167 partial ON1 G gene global sequences with the tips colour coded with the source continent.

As there are far more partial G gene sequences than full genomes, we explored ON1 spatiotemporal patterns using a set of 1,167 global G gene sequences. The global G gene MCC tree is shown in Fig. 3 with the corresponding sampling locations in Supplementary Fig. S1. Viruses from each of the six continents were spread throughout the tree in Fig. 3, i.e. there was neither a single major branch on the tree comprised solely of viruses from a specific continent nor a continent whose viruses were only found within a single major branch, suggesting both intra and inter-continental circulation patterns. However, the majority of the Kilifi ON1 viruses in Fig. 3 clustered with European viruses with a few others clustering with Asian viruses suggesting perhaps a predominantly European source of RSV introductions into Kilifi. Similar to Fig. 2A, the Kilifi viruses in Fig. 3 were placed in multiple (perhaps 4) lineages further supporting the idea of multiple introductions into Kilifi. On the contrary, viruses from each of the remaining two African countries represented (Nigeria and South Africa) were restricted to single major branches even though this could be as a result of the very few ON1 sequences available from these countries (<10 from each). Furthermore, viruses closely related to the ON1 viruses with limited local transmission in Kilifi described above were frequently isolated in other locations.

### 3.4 Genomic diversity of Kilifi RSV-A viruses

**Figure 4.**
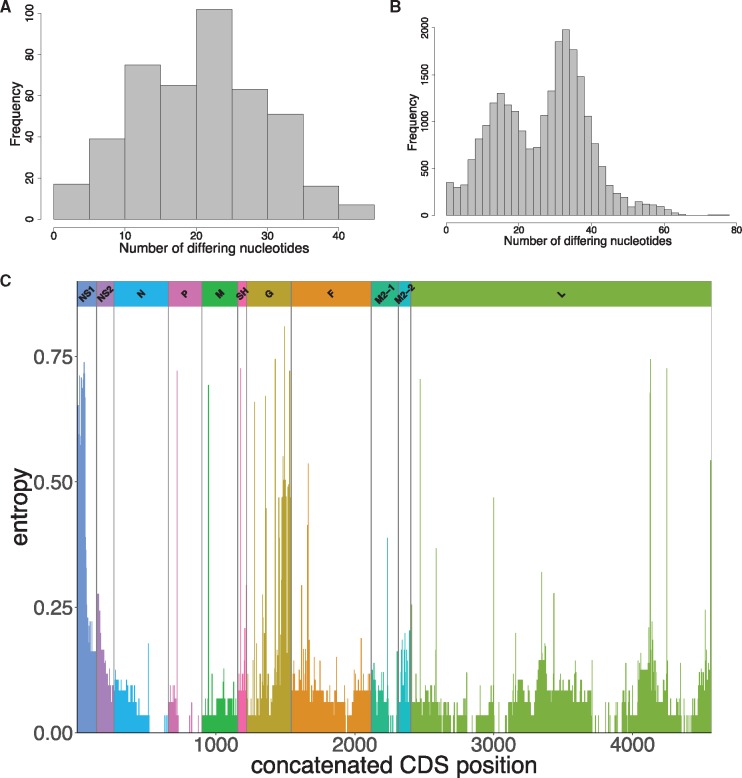
Pairwise genomic distances and genome-wide amino acid variation. The distribution of pairwise genetic distances between genotype GA2 and ON1 genome sequences are shown in (A) and (B), respectively. (C) An entropy plot showing amino acid variation along the concatenated ORFs of Kilifi RSV-A genomes.

Pairwise intra-genotypic genetic diversity analysis of the GA2 and ON1 genomes from Kilifi, Fig. 4A and B, shows unimodal and bimodal distributions, respectively, consistent with two genetically distinct circulating strains of ON1 viruses. Analyzing for substitutions across the genomes by entropy plots (Fig. 4C), we identified 746 SNPs with frequencies of >1% in the set of 184 genomes. Of these SNPs, the majority (589, 78.9%) were found within CDSs. The three CDSs with the most substitutions were the polymerase L (39.6%), the glycoprotein G (14.8%), and the fusion F protein (14.6%). Only 145/589 (24.6%) of these coding mutations resulted in non-synonymous changes, Supplementary Table S3. The majority of the nonsynonymous mutations occurred within the G, SH, and M2-2.

### 3.5 Phylogenetic divergence between ON1 and GA2 viruses

The currently known or *de facto* distinguishing feature of the ON1 from GA2 strains is the 72-nucleotide duplication within the G gene. It has been shown from phylogenetic analysis of the G-gene that RSV-A genotypes form distinct clusters (Peret et al. 1998). However, it has not been investigated if the distinct clustering is replicated in the other genes especially for the closely related genotypes GA2 and ON1 viruses. An exploratory root-to-tip regression analysis of ORF-specific ML trees, whose topologies were similar to the MCC BEAST trees described herein, confirmed that all but the NS1, NS2, and SH proteins had good temporal signals, Supplementary Fig. S3.

**Figure 5.**
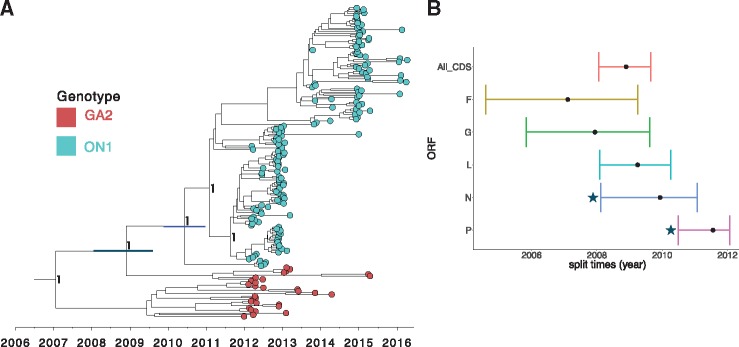
Estimated TMRCA for Kilifi RSV-A viruses and ORFs. (A) MCC tree inferred from 184 RSV-A complete genome sequences (concatenated coding regions only) from Kilifi with the tips colour coded by genotype, i.e. ON1 (cyan) and GA2 (red). The two node bars indicate the 95% HPD interval for the TMRCA for the Kilifi GA2 and ON1 viruses (grey), and Kilifi ON1 strains (blue). Node labels are posterior probabilities indicating support for the selected nodes. (B) The TMRCA (with 95% HPD interval) of the node separating Kilifi RSV-A genotype GA2 and ON1 viruses for a concatenated set of all ORFs and five different ORFs. The stars (*) indicates node posterior support of less than 0.9 (i.e. low support) for the split between GA2 and ON1 in the nucleoprotein (N) and phosphoprotein (P) ORFs.

To assess if the seventy-two–nucleotide duplication is the only marker of the ON1 strains or an accompanying mutation, we analysed the eleven RSV ORFs individually and a concatenated set of ten ORFs (excluding the G). We observed distinct and well-supported ON1 and GA2 clusters in the concatenated set of ten ORFs as well as in five individual coding regions (F, G, L, N, and P), Supplementary Fig. S4, confirming that genetic markers outside G also differentiate the ON1 and GA2 genotypes. The node posterior support, however, for divergence between GA2 and ON1 was quite low (50–70%) in the N and P proteins despite observation of distinct clusters. Nonetheless, determining the order in which the GA2-ON1 divergence in the five ORFs might have occurred was not feasible from this analysis as the divergence could have occurred anywhere on the branch between the GA2-ON1 split time and ON1 TMRCA in Fig. 5A. This divergence chronology dilemma is highlighted by the overlapping MRCA estimates for the individual ORFs of between 2007 and 2011 [95% HPD: 2004.59–2012.06] in Fig. 5B.

### 3.6 Signature substitutions distinguishing ON1 from GA2 viruses

Through a comparative genome-wide scan along the RSV-A coding genome, we analysed for SNPs between the consensus Kilifi ON1 and GA2 viruses. We identified sixty-six signature nucleotide substitutions (defined as SNPs differentiating ON1 from GA2 viruses), Supplementary Table S4. While the majority of these signature substitutions were synonymous, fourteen were nonsynonymous substitutions (Table 1); nine in the G protein, two each in the F and L proteins, and one in the M2-1 protein. However, these signature substitutions had no effect on our RSV multiplex real-time PCR diagnostics as they occur outside the target primer binding sites in the N gene. Changes at the codon sites 142 and 237 of the G protein have previously been shown to characterize antibody escape mutants, and were located within strain-specific epitopes (Martínez, Dopazo, and Melero 1997). The two signature substitutions in the F protein (116 and 122) occur within site p27, which is the most variable antigenic site in the F protein (Hause et al. 2017).

**Table 1.** Signature nonsynonymous substitutions between genotype ON1 and GA2 viruses.

ORF	ORF Nt Pos.^a^	ORF AA Pos.	Nt Change	AA Change	SNP type
G	424	142	TT → CA	L → Q	Substitution
G	622	208	C → A	L → I	Transversion
G	695	232	G → A	G → E	Transition
G	709	237	A → G	N → D	Transition
G	758	253	A → C	K → T	Transversion
G	817	**273**^b^	T → A	Y → N	Transversion
G	821	274	C → T	P → L	Transition
G	851	284	72 nt duplication	24 AA insertion	Deletion
G	929 (GA2: 857)	**310**^b^	C → T	P → L	Transition
G	941 (GA2: 869)	314	T → C	L → P	Transition
F	346	116	A → G	N → D	Transition
F	364	122	G → A	A → T	Transition
M2-1	349	117	A → C	N → H	Transversion
L	1792	598	C → T	H → Y	Transition
L	5175	1725	A → T	E → D	Transversion

a Positions are relative to ON1 strains, in which complementary positions in GA2 (without the duplication) within the G protein are shown in brackets.

b Positively selected sites.

Nt, nucleotide; AA, amino acid; Pos., Position.

### 3.7 Signature substitutions between lineages with successful and limited local transmission

We performed a similar genome-wide comparative scan between the consensus of genomes of viruses with successful (K1 and K2) and those with limited local transmission (K3) for characteristic signature polymorphisms. We identified thirty-three SNPs between these two groups of viruses, Supplementary Table S5, of which nine resulted in nonsynonymous changes; five in G, two in F, and one each in M2-2 and L. In three of these nine nonsynonymous SNPs, the K3 viruses shared substitutions with the GA2 viruses (G: codons P274L and P310L, and F: codon A122T). Whether these polymorphisms are neutral mutations or influence local transmission of the virus warrants further investigation.

### 3.8 Patterns of selective pressure

We conducted selection analysis on all eleven RSV ORFs for the dataset, Supplementary Table S6. ORF-wide episodic diversifying selection was only detected in the NS1 and M proteins. A total of nine positively selected codon sites were identified within the G (73, 201, 250, 251, 273, 310), NS2 (15), and the L (2030, 2122) by at least one method, with site 310 in the G identified as positively selected by all the four methods. Notably, sites 273 and 310 (shown in bold in Table 1) within the G protein detected to be under positive selection were also identified as signature SNPs. However, the number of positively selected sites could have been underestimated in the analysis that was limited to Kilifi RSV-A genomes and care should be taken while interpreting these results as some of the positively selected sites were only detected by one method and at default (less stringent) cut-offs.

## 4. Discussion

Here we report an in-depth analysis of local and global RSV genotype ON1 evolution and transmission using WGS data. We describe RSV-A genomic diversity and identify polymorphisms with the most potential in influencing RSV evolution and phenotype. Utilizing genomes from samples collected between 2010 and 2016, including 184 complete genomes from Kilifi alone, we obtained a finer resolution on the pattern of RSV introductions, persistence and evolution in a defined location, and the changes within the genome that might be important for the persistent circulation of the virus.

Genetic variation not only provides important insights into RSV relatedness by which to infer transmission events but also highlights potential functional changes in the virus. From our analysis, we find that substitutions are widespread across the RSV genome but occur at higher frequency within the structural proteins (G and F) and in parts of the polymerase (L). The G protein has the most genetic flexibility of the RSV ORFs to accommodate frequent substitutions including large duplications, and previous studies have described epitope positions associated with escape using specific monoclonal antibodies or in natural isolates (García et al. 1994; Cane and Pringle 1995; Cane 1997; Martínez, Dopazo, and Melero 1997). The F protein site p27 with two signature substitutions has been shown to possess greater binding affinity for serum antibodies from young children (<2 years) than any of the other antigenic sites in the F protein and may be responsible for group specific immunity that distinguish between RSV-A and RSV-B viruses (Fuentes et al. 2016). The implications of observed substitutions in the L protein of the ON1 viruses remain unclear. However, considering both its role in genome replication and the emergence of the seventy-two–nucleotide duplication in the G ORF, we posit that either (1) these polymorphisms might have resulted in a sloppy polymerase, that resulted in a slip that generated the seventy-two–nucleotide duplication in the G ORF (Komissarova and Kashlev 1997), or (2) the seventy-two–nucleotide duplication in the G may present a metabolic challenge for replicating a large genome and thereby facilitate adaptive polymorphisms within the polymerase (Canchaya et al. 2003). While we also found a considerable number of SNPs in ORFs other than the G, F, and L proteins, only a very minor proportion of those changes resulted in amino acid substitutions implying very strong purifying selection.

Based on distinct phylogenetic clustering of ON1 and GA2 viruses in five ORFs, the emergence of ON1 is characterized by additional substitutions across the genome in addition to the seventy-two–nucleotide duplication within the G gene. However, assuming ON1 diverged from GA2 and through a single ancestral virus, it is unclear whether the multiple signature substitutions differentiating ON1 from GA2 viruses all arose from that single split event or have been acquired progressively over time. In case of the latter, the chronology of changes across the different ORFs is unclear. Understanding how and which mutations define the emergence of a new RSV variant may be important in describing substitutions that are either crucial for the survival of the variant and/or of some complementary structural or functional integrity. It is also likely that some of these substitutions are nothing more than genetic hitchhikers. Notwithstanding this lack of clarity on ON1 emergence, it has been shown for influenza A viruses that linked selection amongst antigenic and non-antigenic genes influences the evolutionary dynamics of novel antigenic variants (Raghwani, Thompson, and Koelle 2017). Further, it has been demonstrated experimentally that adaptive evolution is a multi-step process that occurs in waves (Stern et al. 2017). The initial adaptive wave is thought to occur rapidly and is characterized by founder or gatekeeper mutations. Thereafter, additional waves of evolutionary fine-tuning occur (Grubaugh and Andersen 2017). Similar studies in RSV would be important to determine if such dynamics do characterize RSV’s evolutionary history and may also inform the design of an RSV vaccine.

ON1 is rapidly replacing GA2 in Kilifi, suggesting that this variant may have some fitness advantage in this location. We have however previously showed that genotype ON1 viruses did not result in more severe disease compared to GA2 viruses in Kilifi (Otieno et al. 2017). Globally, ON1 prevalence varies by location and there are conflicting reports with regards to differences in virulence between ON1 and GA2 strains (Panayiotou et al. 2014; Yoshihara et al. 2016). Even with the discordant results, which may be due to differences in study populations and analysis methods, there might be phenotypic differences between viruses belonging to these two genotypes. Identification of such phenotypic differences and the potential drivers might augment our current understanding of the pathogenesis of this virus. Expanded RSV surveillance in additional locations will offer better insight into the nature of these replacement dynamics.

Observations from this study using whole genomes reinforce previous findings based on partial G-gene sequences (Agoti et al. 2014b, 2015b; Otieno et al. 2016, 2017) that RSV epidemics are characterized by the introduction and circulation of multiple variants. In addition, persistence within the community seems to be sustained by only a proportion of these introductions. We have characterized genomic substitutions that distinguish between successful and dead-end ON1 introductions in Kilifi. Nonetheless, it is evident that besides viral genetic factors there could be other determinants of successful onward transmission of a virus lineage. ON1 strains that were nonpersistent in Kilifi were abundant in other parts of the world albeit with varied frequencies relative to other genotypes. Such determinants could include the host factors (e.g. births, immunity, genetics, contact patterns, and mobility) and environmental factors (e.g. temperature, rainfall, and humidity) which warrant further investigations.

We live in times of rapid global movement of people, which may influence the spread of infectious diseases. The observation that most of the Kilifi sequences clustered with sequences from Europe and Asia suggests that RSV introductions into Kilifi originate predominantly from these two continents. It might not be surprising that Europe could be a source of RSV introduction into Kilifi, or a destination for viruses from Kilifi, considering that it accounts for the largest single group of tourists to Kenya (The Report: Kenya 2017 2017). In addition, the increasing Chinese economic interests in Africa (including Kenya) has resulted in an influx of Chinese into Africa for trade, work and tourism (More than minerals) and may account for the Asia-like ON1 strains. However, there are far too few partial ON1 sequences from Africa (only from Kenya, South Africa, and Nigeria) and no ON1 genomes from outside Kilifi Kenya to help define intra-African transmission dynamics in detail. In fact, a recent study suggests that domestic tourism accounts for more than half of the growth in Kenya’s tourism (Sunday 2018). As such, availability of sequences from across the country would be critical in deciphering if and how such tourist activities influence virus transmission patterns in Kenya. Such studies could be helpful in the design of future RSV transmission intervention strategies.

**Conflict of interest:** None declared.

## Supplementary Material

Supplementary Figures

Supplementary S1 Table

Supplementary S2 Table

Supplementary S3 Table

Supplementary S4 Table

Supplementary S5 Table

Supplementary S6 Table

Supporting Information Legends
