# Whole genome analysis of local Kenyan and global sequences unravels the epidemiological and molecular evolutionary dynamics of RSV genotype ON1 strains

**DOI:** 10.1093/ve/vey036

**Published:** 2018-11-16

**Authors:** J R Otieno, E M Kamau, J W Oketch, J M Ngoi, A M Gichuki, Š Binter, G P Otieno, M Ngama, C N Agoti, P A Cane, P Kellam, M Cotten, P Lemey, D J Nokes

*Virus Evolution*, Volume 4, Issue 2, 1 July 2018, vey027 https://doi.org/10.1093/ve/vey027

Published: 24 September 2018

Upon initial publication this article omitted funding information and an acknowledgements section. The article has been updated to reflect that funding is supplied by Wellcome Trust (grant ref: 102975) and an acknowledgements section has been added.

