# Supplementary material for: Whole genome analysis of local Kenyan and global sequences unravels the epidemiological and molecular evolutionary dynamics of RSV genotype ON1 strains
Source: Virus Evol. 2018 Sep 24;4(2):vey027. doi: 10.1093/ve/vey027 (PMC6153471; doi:10.1093/ve/vey027)
Supplement: Supplementary Figures [file vey027_supplementary_figures.pdf]

S1 Fig: *Sampling locations of the global ON1 G-gene dataset with circles representative of relative proportion of contributing sequences by country*

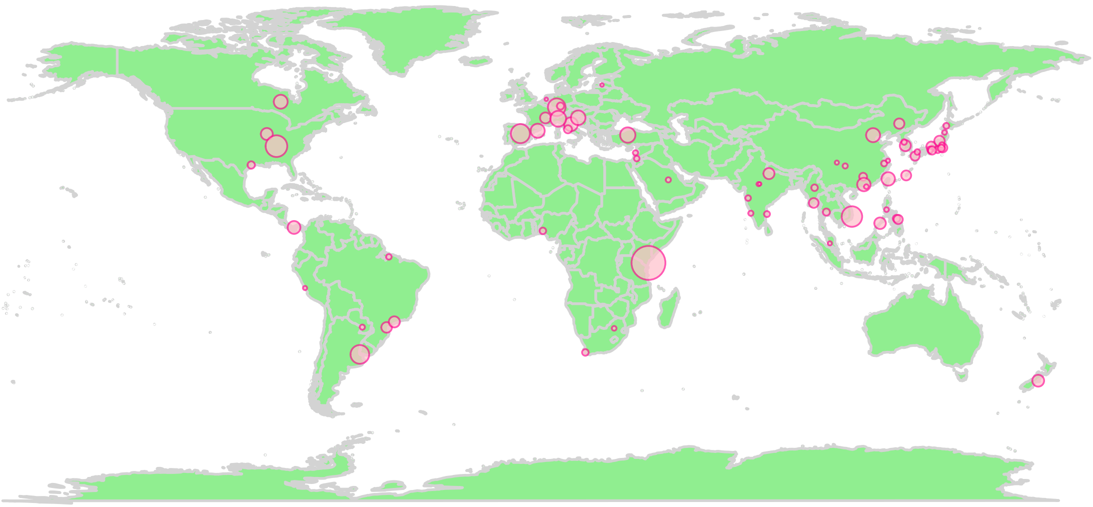

S2 Fig: *Histograms showing distribution of Ct values for samples from the (A) KHDSS and (B) KCH*

A.

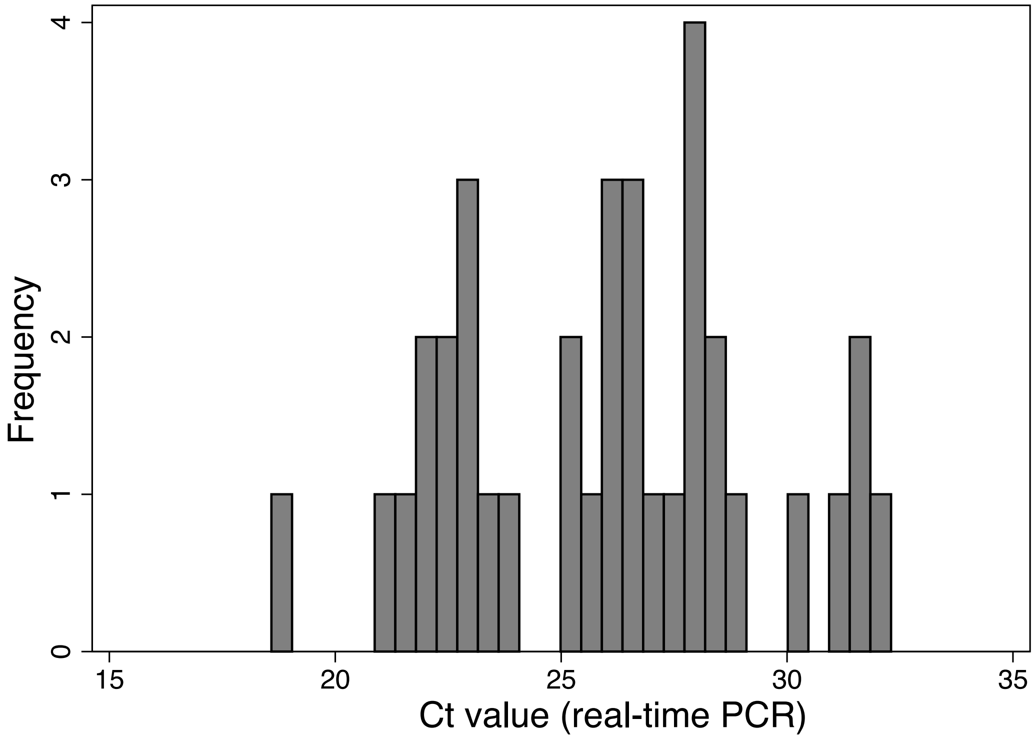

B.

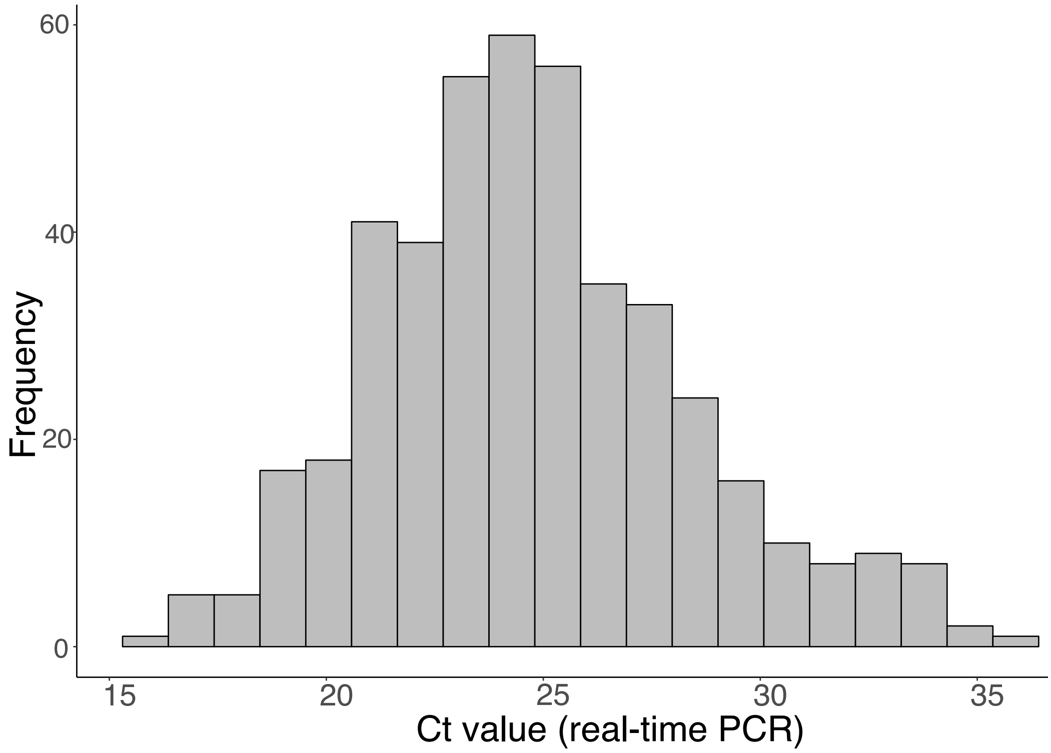

S3 Fig: *Root-to-tip regression analysis of Kilifi RSV-A ORFs*

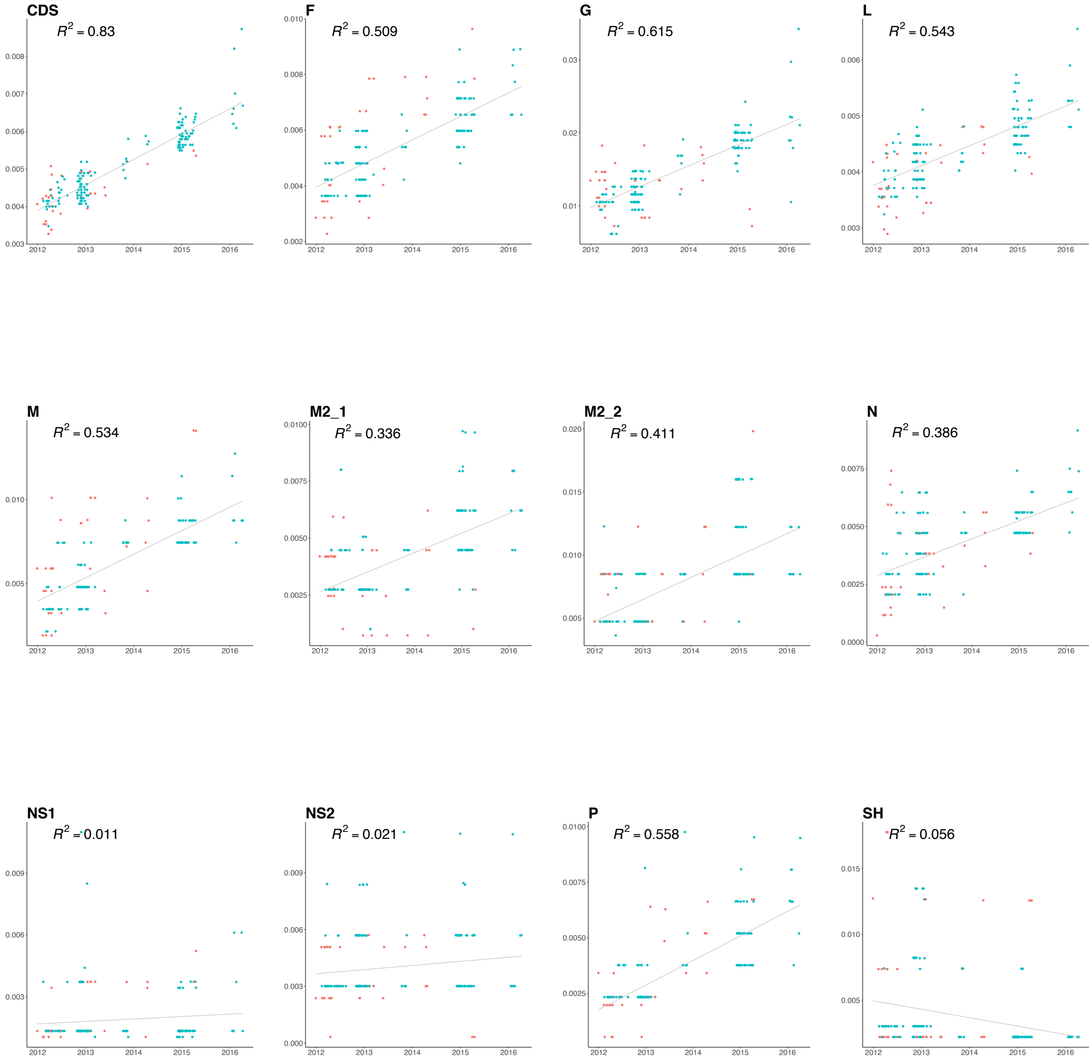

S4 Fig: *BEAST MCC trees showing divergence between ON1(cyan) and GA2 (red) ORFs*

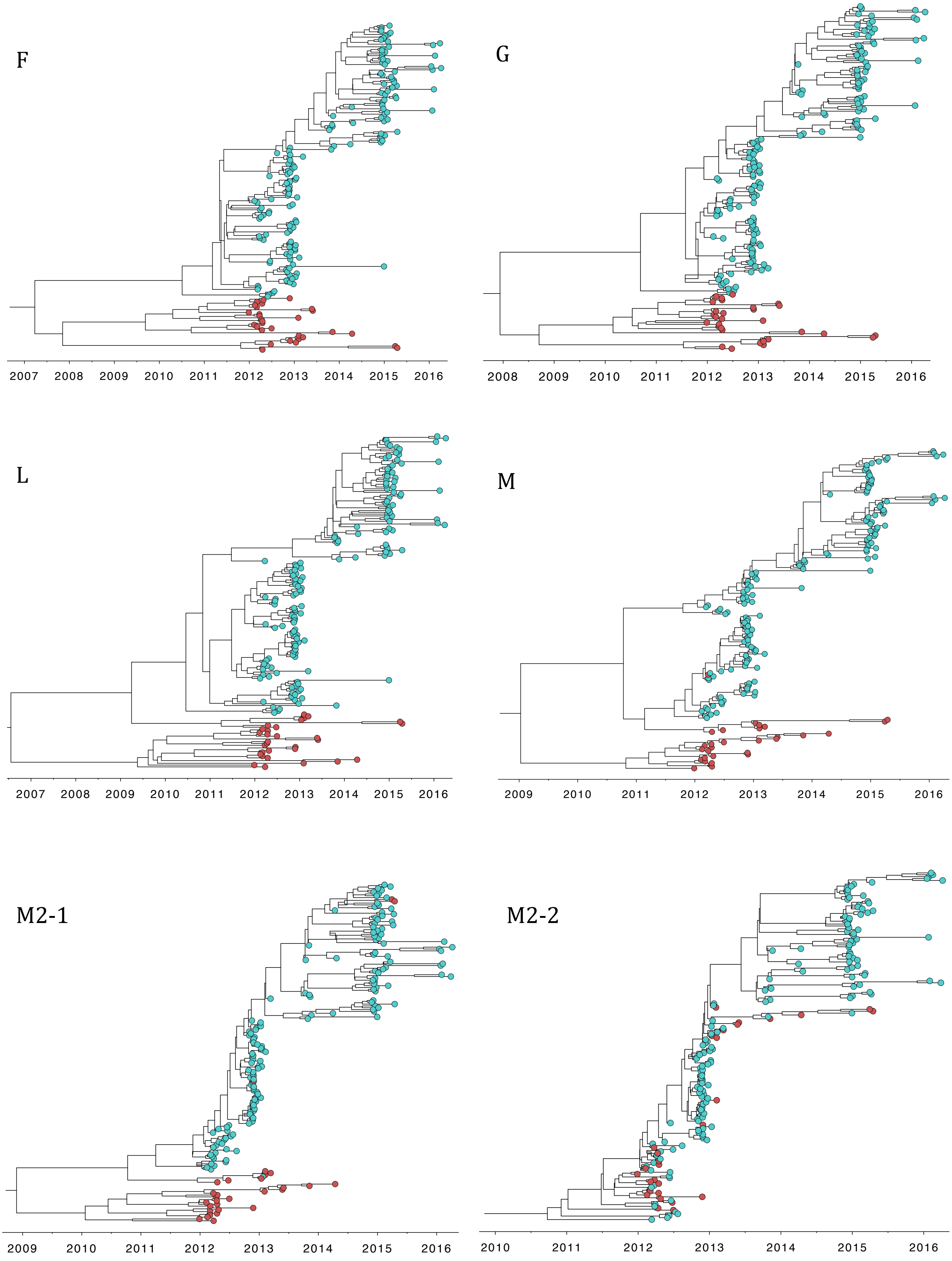

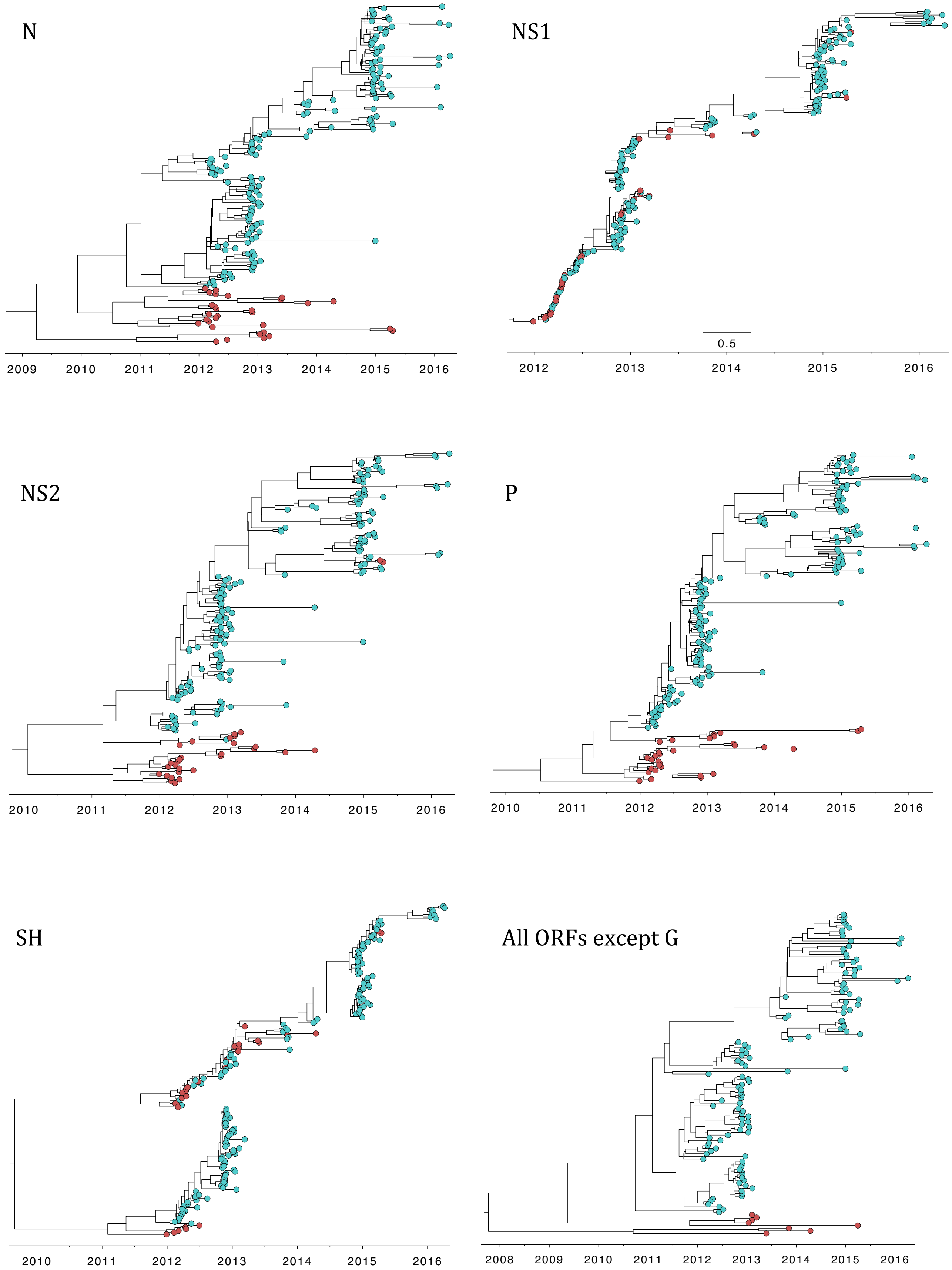
N
2009 2010 2011 2012 2013 2014 2015 2016
NS1
0.5
2012 2013 2014 2015 2016
NS2
2010 2011 2012 2013 2014 2015 2016
P
2010 2011 2012 2013 2014 2015 2016
SH
2010 2011 2012 2013 2014 2015 2016
All ORFs except G
2008 2009 2010 2011 2012 2013 2014 2015 2016
