# Supplementary material for: Whole genome analysis of local Kenyan and global sequences unravels the epidemiological and molecular evolutionary dynamics of RSV genotype ON1 strains
Source: Virus Evol. 2018 Sep 24;4(2):vey027. doi: 10.1093/ve/vey027 (PMC6153471; doi:10.1093/ve/vey027)
Supplement: Supplementary S1 Table [file vey027_supplementary_s1_table.pdf]

**Clock + Population model**	**PS**	**SS**
Strict + Skyline	-40221.86	-40237.00
Relaxed + Skyline	-40203.78	-40212.06
Strict + Exponential	-40285.47	-40293.39
Strict + Constant	-40284.81	-40293.96
Relaxed + Constant	-40263.39	-40275.27
Relaxed + Exponential	-40272.20	-40283.01
