# Supplementary material for: Whole genome analysis of local Kenyan and global sequences unravels the epidemiological and molecular evolutionary dynamics of RSV genotype ON1 strains
Source: Virus Evol. 2018 Sep 24;4(2):vey027. doi: 10.1093/ve/vey027 (PMC6153471; doi:10.1093/ve/vey027)
Supplement: Supplementary S2 Table [file vey027_supplementary_s2_table.pdf]

Sample	Genome_Accession	genotype	PCR_strategy	No_of_Reads	RSV_Reads	Assembly_length	mean_coverage	Assembler	RT_PCR_Ct	collection_date	SRA_Accession
KEN/Kilifi/WGS/1022_28/12/2011	MH181878	GA2	6-amplicon	1,270,622	393,958	14,912	4,483.13	VIRALNGS	NA	28/12/11	SAMN08724833
KEN/Kilifi/WGS/1024_09/02/2012	MH181879	GA2	6-amplicon	1,407,764	270,288	14,697	2,961.69	VIRALNGS	22.94	09/02/12	SAMN08724834
KEN/Kilifi/WGS/1025_13/02/2012	MH181908	ON1	14-amplicon	3,293,722	3,088,934	15,168	53,178.19	VIRALNGS	19.26	13/02/12	SAMN08724835
KEN/Kilifi/WGS/1026_16/02/2012	MH181880	GA2	6-amplicon	1,454,390	332,690	14,697	3,457.63	VIRALNGS	21.38	16/02/12	SAMN08724836
KEN/Kilifi/WGS/1028_02/03/2012	MH181881	GA2	6-amplicon	939,372	442,216	14,913	4,740.40	VIRALNGS	19.55	02/03/12	SAMN08724837
KEN/Kilifi/WGS/1029_03/03/2012	MH181882	GA2	6-amplicon	1,125,168	465,202	14,903	5,023.98	VIRALNGS	21.31	03/03/12	SAMN08724838
KEN/Kilifi/WGS/1031_05/03/2012	MH181883	GA2	6-amplicon	1,385,586	1,036,466	14,915	11,068.56	VIRALNGS	20.17	05/03/12	SAMN08724839
KEN/Kilifi/WGS/1032_07/03/2012	MH181909	ON1	14-amplicon	2,602,100	2,252,496	15,070	38,777.92	VIRALNGS	21.64	07/03/12	SAMN08724840
KEN/Kilifi/WGS/1033_09/03/2012	MH181910	ON1	14-amplicon	3,393,018	3,272,224	15,197	55,740.25	VIRALNGS	17.75	09/03/12	SAMN08724841
KEN/Kilifi/WGS/1034_11/03/2012	MH181911	ON1	14-amplicon	2,218,728	1,065,438	14,959	18,236.55	VIRALNGS	24.22	11/03/12	SAMN08724842
KEN/Kilifi/WGS/1035_16/03/2012	MH181912	ON1	14-amplicon	3,380,044	3,262,410	15,180	55,995.24	VIRALNGS	16.98	16/03/12	SAMN08724843
KEN/Kilifi/WGS/1036_21/03/2012	MH181913	ON1	14-amplicon	3,858,536	3,746,792	15,205	64,113.11	VIRALNGS	17.98	21/03/12	SAMN08724844
KEN/Kilifi/WGS/1037_23/03/2012	MH181884	GA2	6-amplicon	984,444	1,626	14,332	44.33	VIRALNGS	24.83	23/03/12	SAMN08724845
KEN/Kilifi/WGS/1038_23/03/2012	MH181914	ON1	14-amplicon	3,886,448	3,641,296	15,152	62,377.42	VIRALNGS	20.41	23/03/12	SAMN08724846
KEN/Kilifi/WGS/1039_25/03/2012	MH181885	GA2	6-amplicon	1,620,128	33,722	14,686	406.46	VIRALNGS	26.31	25/03/12	SAMN08724847
KEN/Kilifi/WGS/1040_26/03/2012	MH181915	ON1	14-amplicon	4,350,992	3,963,646	15,061	66,457.67	VIRALNGS	20.16	26/03/12	SAMN08724848
KEN/Kilifi/WGS/1041_29/03/2012	MH181916	ON1	14-amplicon	3,056,872	2,224,280	15,140	38,552.58	VIRALNGS	21.25	29/03/12	SAMN08724849
KEN/Kilifi/WGS/1043_05/04/2012	MH181917	ON1	14-amplicon	2,191,722	1,876,406	15,232	32,307.51	SPADES	24.16	05/04/12	SAMN08724850
KEN/Kilifi/WGS/1044_10/04/2012	MH181918	ON1	14-amplicon	2,916,484	2,762,130	15,164	47,482.85	VIRALNGS	20.87	10/04/12	SAMN08724851
KEN/Kilifi/WGS/1045_10/04/2012	MH181886	GA2	6-amplicon	1,494,678	958,672	14,891	9,996.56	VIRALNGS	22.16	10/04/12	SAMN08724852
KEN/Kilifi/WGS/1046_13/04/2012	MH181887	GA2	6-amplicon	1,251,788	880,908	14,549	9,824.38	VIRALNGS	21.33	13/04/12	SAMN08724853
KEN/Kilifi/WGS/1047_15/04/2012	MH181888	GA2	6-amplicon	1,641,672	383,808	14,697	4,419.82	VIRALNGS	22.62	15/04/12	SAMN08724854
KEN/Kilifi/WGS/1048_16/04/2012	MH181889	GA2	6-amplicon	1,345,204	269,926	14,669	3,341.61	VIRALNGS	25.2	16/04/12	SAMN08724855
KEN/Kilifi/WGS/1049_17/04/2012	MH181890	GA2	6-amplicon	1,287,742	912,588	14,876	9,912.84	VIRALNGS	20.66	17/04/12	SAMN08724856
KEN/Kilifi/WGS/1050_17/04/2012	MH181891	GA2	6-amplicon	1,483,678	35,648	14,589	431.77	VIRALNGS	25.41	17/04/12	SAMN08724857
KEN/Kilifi/WGS/1051_24/04/2012	MH181919	ON1	14-amplicon	3,036,992	2,853,918	15,198	49,164.32	VIRALNGS	18.44	24/04/12	SAMN08724858
KEN/Kilifi/WGS/1052_25/04/2012	MH181892	GA2	6-amplicon	1,555,690	879,362	14,693	9,480.92	VIRALNGS	19.98	25/04/12	SAMN08724859
KEN/Kilifi/WGS/1053_26/04/2012	MH181920	ON1	14-amplicon	2,552,538	2,084,818	15,116	35,963.84	VIRALNGS	21.81	26/04/12	SAMN08724860
KEN/Kilifi/WGS/1054_16/05/2012	MH181921	ON1	14-amplicon	3,235,132	2,694,000	15,138	45,968.25	VIRALNGS	20.81	16/05/12	SAMN08724861
KEN/Kilifi/WGS/1056_30/05/2012	MH181922	ON1	14-amplicon	2,935,820	1,780,478	15,049	30,814.75	VIRALNGS	22.72	30/05/12	SAMN08724862
KEN/Kilifi/WGS/1058_08/06/2012	MH181923	ON1	14-amplicon	2,312,598	1,906,202	15,106	32,807.75	SPADES	21.04	08/06/12	SAMN08724863
KEN/Kilifi/WGS/1059_08/06/2012	MH181924	ON1	14-amplicon	2,225,810	2,187,994	15,215	37,245.25	VIRALNGS	16.86	08/06/12	SAMN08724864
KEN/Kilifi/WGS/1060_11/06/2012	MH181925	ON1	14-amplicon	2,470,026	2,348,036	15,188	40,483.39	VIRALNGS	18.23	11/06/12	SAMN08724865
KEN/Kilifi/WGS/1061_13/06/2012	MH181926	ON1	14-amplicon	2,071,626	1,772,624	15,049	30,725.88	VIRALNGS	21.53	13/06/12	SAMN08724866
KEN/Kilifi/WGS/1062_15/06/2012	MH181927	ON1	14-amplicon	1,688,810	1,123,748	15,059	19,373.45	VIRALNGS	23.13	15/06/12	SAMN08724867
KEN/Kilifi/WGS/1063_18/06/2012	MH181928	ON1	14-amplicon	2,887,012	2,207,944	15,042	37,782.03	VIRALNGS	21.58	18/06/12	SAMN08724868
KEN/Kilifi/WGS/1064_24/06/2012	MH181893	GA2	6-amplicon	1,151,112	400,912	14,572	4,631.14	VIRALNGS	23.79	24/06/12	SAMN08724869
KEN/Kilifi/WGS/1066_28/06/2012	MH181929	ON1	14-amplicon	2,729,430	2,628,610	15,175	45,411.89	VIRALNGS	18.55	28/06/12	SAMN08724870
KEN/Kilifi/WGS/1067_30/06/2012	MH181894	GA2	6-amplicon	1,691,236	114,412	14,698	1,384.07	VIRALNGS	29.82	30/06/12	SAMN08724871
KEN/Kilifi/WGS/1068_10/07/2012	MH181930	ON1	14-amplicon	2,525,642	2,383,726	15,207	40,719.45	VIRALNGS	20.98	10/07/12	SAMN08724872
KEN/Kilifi/WGS/1070_23/07/2012	MH181931	ON1	14-amplicon	2,159,538	1,535,640	15,049	26,480.83	VIRALNGS	25.32	23/07/12	SAMN08724873
KEN/Kilifi/WGS/1071_14/08/2012	MH181932	ON1	14-amplicon	2,675,604	2,520,028	15,233	43,447.66	SPADES	21.45	14/08/12	SAMN08724874
KEN/Kilifi/WGS/1075_27/10/2012	MH181933	ON1	14-amplicon	2,194,346	1,857,942	15,232	31,956.04	SPADES	24.8	27/10/12	SAMN08724875
KEN/Kilifi/WGS/1076_30/10/2012	MH181934	ON1	14-amplicon	3,041,964	2,314,252	15,068	39,401.72	VIRALNGS	23.24	30/10/12	SAMN08724876
KEN/Kilifi/WGS/1077_31/10/2012	MH181935	ON1	14-amplicon	2,438,426	2,175,418	15,232	37,609.41	SPADES	22.49	31/10/12	SAMN08724877
KEN/Kilifi/WGS/1078_31/10/2012	MH181936	ON1	14-amplicon	2,476,744	2,311,046	15,230	39,698.20	SPADES	21.93	31/10/12	SAMN08724878
KEN/Kilifi/WGS/1079_05/11/2012	MH181937	ON1	14-amplicon	2,114,774	1,819,602	15,232	31,236.65	SPADES	22.13	05/11/12	SAMN08724879
KEN/Kilifi/WGS/1080_05/11/2012	MH181938	ON1	14-amplicon	1,786,686	1,465,770	15,074	25,257.87	VIRALNGS	25.1	05/11/12	SAMN08724880
KEN/Kilifi/WGS/1081_07/11/2012	MH181939	ON1	14-amplicon	2,096,870	1,926,670	15,151	33,319.15	VIRALNGS	21.73	07/11/12	SAMN08724881
KEN/Kilifi/WGS/1082_10/11/2012	MH181940	ON1	14-amplicon	1,699,646	1,240,272	15,214	21,458.29	SPADES	24.24	10/11/12	SAMN08724882
KEN/Kilifi/WGS/1083_12/11/2012	MH181941	ON1	14-amplicon	2,060,860	1,899,520	15,222	32,687.98	SPADES	19.74	12/11/12	SAMN08724883
KEN/Kilifi/WGS/1084_15/11/2012	MH181942	ON1	14-amplicon	1,632,392	380,376	14,959	6,498.76	VIRALNGS	26.16	15/11/12	SAMN08724884
KEN/Kilifi/WGS/1086_15/11/2012	MH181943	ON1	14-amplicon	2,275,828	2,057,472	15,226	35,609.99	SPADES	25.78	15/11/12	SAMN08724885
KEN/Kilifi/WGS/1087_17/11/2012	MH181944	ON1	14-amplicon	2,287,906	2,032,176	15,049	34,971.14	VIRALNGS	21.43	17/11/12	SAMN08724886
KEN/Kilifi/WGS/1088_19/11/2012	MH181945	ON1	14-amplicon	2,701,940	2,645,394	15,171	45,076.37	VIRALNGS	18.92	19/11/12	SAMN08724887
KEN/Kilifi/WGS/1089_19/11/2012	MH181946	ON1	14-amplicon	2,355,836	1,933,388	15,110	33,278.48	VIRALNGS	23.96	19/11/12	SAMN08724888
KEN/Kilifi/WGS/1090_19/11/2012	MH181947	ON1	14-amplicon	2,286,976	2,185,794	15,171	37,746.72	VIRALNGS	18.54	19/11/12	SAMN08724889
KEN/Kilifi/WGS/1091_22/11/2012	MH181948	ON1	14-amplicon	2,359,658	2,288,890	15,198	39,369.58	VIRALNGS	16.91	22/11/12	SAMN08724890
KEN/Kilifi/WGS/1092_23/11/2012	MH181949	ON1	14-amplicon	2,060,978	1,566,076	15,025	26,801.24	VIRALNGS	23.19	23/11/12	SAMN08724891
KEN/Kilifi/WGS/1093_23/11/2012	MH181950	ON1	14-amplicon	1,182,120	416,874	15,150	7,324.31	SPADES	30.01	23/11/12	SAMN08724892
KEN/Kilifi/WGS/1094_23/11/2012	MH181951	ON1	14-amplicon	2,346,184	2,265,498	15,227	39,009.62	SPADES	17.63	23/11/12	SAMN08724893
KEN/Kilifi/WGS/1095_24/11/2012	MH181952	ON1	14-amplicon	2,169,590	2,002,620	15,104	34,587.63	VIRALNGS	24.14	24/11/12	SAMN08724894
KEN/Kilifi/WGS/1096_24/11/2012	MH181953	ON1	14-amplicon	2,697,992	2,627,426	15,183	45,492.81	VIRALNGS	19.92	24/11/12	SAMN08724895
KEN/Kilifi/WGS/1097_24/11/2012	MH181954	ON1	14-amplicon	2,698,368	2,339,798	15,231	40,334.68	SPADES	21.98	24/11/12	SAMN08724896
KEN/Kilifi/WGS/1098_25/11/2012	MH181955	ON1	14-amplicon	2,671,940	2,502,752	15,232	43,168.08	SPADES	23.48	25/11/12	SAMN08724897
KEN/Kilifi/WGS/1099_25/11/2012	MH181956	ON1	14-amplicon	2,654,944	2,542,430	15,183	43,877.84	VIRALNGS	20.64	25/11/12	SAMN08724898
KEN/Kilifi/WGS/1100_25/11/2012	MH181957	ON1	14-amplicon	2,232,292	2,011,214	15,033	34,492.22	VIRALNGS	21.98	25/11/12	SAMN08724899
KEN/Kilifi/WGS/1101_25/11/2012	MH181895	GA2	6-amplicon	1,476,584	531,814	14,883	6,072.07	VIRALNGS	24.75	25/11/12	SAMN08724900
KEN/Kilifi/WGS/1102_27/11/2012	MH181958	ON1	14-amplicon	3,137,122	3,001,672	15,185	51,523.50	VIRALNGS	20.18	27/11/12	SAMN08724901
KEN/Kilifi/WGS/1103_27/11/2012	MH181959	ON1	14-amplicon	2,561,480	2,387,270	15,145	41,078.44	VIRALNGS	20.05	27/11/12	SAMN08724902
KEN/Kilifi/WGS/1104_27/11/2012	MH181960	ON1	14-amplicon	2,367,006	2,217,710	15,139	38,181.27	VIRALNGS	22.13	27/11/12	SAMN08724903
KEN/Kilifi/WGS/1105_28/11/2012	MH181961	ON1	14-amplicon	2,553,394	2,370,212	15,192	40,462.29	SPADES	21.72	28/11/12	SAMN08724904
KEN/Kilifi/WGS/1106_28/11/2012	MH181962	ON1	14-amplicon	2,958,126	2,940,148	15,176	50,168.19	VIRALNGS	18.98	28/11/12	SAMN08724905
KEN/Kilifi/WGS/1107_28/11/2012	MH181963	ON1	14-amplicon	2,781,054	1,786,998	15,030	31,236.67	VIRALNGS	27.6	28/11/12	SAMN08724906
KEN/Kilifi/WGS/1108_28/11/2012	MH181896	GA2	6-amplicon	1,553,924	611,370	14,949	7,511.50	VIRALNGS	25.74	28/11/12	SAMN08724907
KEN/Kilifi/WGS/1109_29/11/2012	MH181964	ON1	14-amplicon	3,244,876	3,065,026	15,232	52,644.38	SPADES	27.01	29/11/12	SAMN08724908
KEN/Kilifi/WGS/1110_29/11/2012	MH181965	ON1	14-amplicon	2,750,196	2,413,804	15,147	41,452.34	VIRALNGS	22.03	29/11/12	SAMN08724909
KEN/Kilifi/WGS/1112_29/11/2012	MH181966	ON1	14-amplicon	3,284,922	2,341,218	15,049	40,716.26	VIRALNGS	28.78	29/11/12	SAMN08724910
KEN/Kilifi/WGS/1113_03/12/2012	MH181967	ON1	14-amplicon	3,211,012	3,101,106	15,228	53,002.60	SPADES	21.08	03/12/12	SAMN08724911
KEN/Kilifi/WGS/1114_03/12/2012	MH181968	ON1	14-amplicon	2,226,714	1,582,202	15,050	28,471.78	VIRALNGS	27.02	03/12/12	SAMN08724912
KEN/Kilifi/WGS/1115_04/12/2012	MH181969	ON1	14-amplicon	2,465,632	1,805,348	15,082	31,377.33	VIRALNGS	25	04/12/12	SAMN08724913
KEN/Kilifi/WGS/1117_10/12/2012	MH181970	ON1	14-amplicon	2,254,784	1,873,994	15,232	32,492.81	SPADES	22.1	10/12/12	SAMN08724914
KEN/Kilifi/WGS/1118_12/12/2012	MH181971	ON1	14-amplicon	2,783,040	2,095,178	15,049	36,339.50	VIRALNGS	22.79	12/12/12	SAMN08724915
KEN/Kilifi/WGS/1119_15/12/2012	MH181972	ON1	14-amplicon	2,354,872	2,156,166	15,152	37,309.87	VIRALNGS	20.03	15/12/12	SAMN08724916
KEN/Kilifi/WGS/1120_19/12/2012	MH181973	ON1	14-amplicon	2,108,962	1,920,920	15,099	32,979.37	VIRALNGS	19.74	19/12/12	SAMN08724917
KEN/Kilifi/WGS/1121_21/12/2012	MH181974	ON1	14-amplicon	3,135,066	2,966,696	15,170	51,695.42	VIRALNGS	20.48	21/12/12	SAMN08724918
KEN/Kilifi/WGS/1122_23/12/2012	MH181975	ON1	14-amplicon	2,404,842	2,248,908	15,145	38,581.46	VIRALNGS	18.57	23/12/12	SAMN08724919
KEN/Kilifi/WGS/1124_24/12/2012	MH181976	ON1	14-amplicon	1,571,860	798,782	15,049	13,968.73	VIRALNGS	23.75	24/12/12	SAMN08724920
KEN/Kilifi/WGS/1125_26/12/2012	MH181977	ON1	14-amplicon	2,626,156	2,516,622	15,227	43,345.79	SPADES	16.88	26/12/12	SAMN08724921
KEN/Kilifi/WGS/1126_03/01/2013	MH181978	ON1	14-amplicon	1,969,924	1,436,248	14,959	24,636.93	VIRALNGS	23.18	03/01/13	SAMN08724922
KEN/Kilifi/WGS/1128_07/01/2013	MH181979	ON1	14-amplicon	1,733,862	1,428,270	15,075	24,776.24	VIRALNGS	23.35	07/01/13	SAMN08724923
KEN/Kilifi/WGS/1129_08/01/2013	MH181980	ON1	14-amplicon	2,637,728	2,557,822	15,169	43,864.26	VIRALNGS	20.43	08/01/13	SAMN08724924
KEN/Kilifi/WGS/1131_10/01/2013	MH181981	ON1	14-amplicon	2,778,546	2,598,338	15,114	44,738.50	VIRALNGS	26.33	10/01/13	SAMN08724925
KEN/Kilifi/WGS/1132_11/01/2013	MH181982	ON1	14-amplicon	2,898,380	2,801,846	15,322	48,134.24	SPADES	19.8	11/01/13	SAMN08724926
KEN/Kilifi/WGS/1133_11/01/2013	MH181983	ON1	14-amplicon	3,266,658	3,046,788	15,150	52,451.71	VIRALNGS	22.94	11/01/13	SAMN08724927
KEN/Kilifi/WGS/1134_12/01/2013	MH181984	ON1	14-amplicon	2,704,858	2,439,372	15,118	41,877.57	VIRALNGS	22.01	12/01/13	SAMN08724928
KEN/Kilifi/WGS/1138_14/01/2013	MH181897	GA2	6-amplicon	1,980,492	1,268,738	14,985	15,156.29	VIRALNGS	20.06	14/01/13	SAMN08724929
KEN/Kilifi/WGS/1140_17/01/2013	MH181985	ON1	14-amplicon	2,211,052	1,873,618	15,123	32,450.84	VIRALNGS	21.47	17/01/13	SAMN08724930
KEN/Kilifi/WGS/1141_17/01/2013	MH181986	ON1	14-amplicon	2,438,632	2,291,448	15,160	39,710.23	VIRALNGS	22.23	17/01/13	SAMN08724931
KEN/Kilifi/WGS/1142_21/01/2013	MH181987	ON1	14-amplicon	2,399,228	2,032,498	15,136	35,144.81	VIRALNGS	23.53	21/01/13	SAMN08724932
KEN/Kilifi/WGS/1144_24/01/2013	MH181988	ON1	6-amplicon	996,732	167,134	14,906	2,385.06	VIRALNGS	20.97	24/01/13	SAMN08724933
KEN/Kilifi/WGS/1146_03/02/2013	MH181898	GA2	6-amplicon	1,460,748	247,094	14,693	2,739.55	VIRALNGS	19.26	03/02/13	SAMN08724934
KEN/Kilifi/WGS/1148_07/02/2013	MH181899	GA2	6-amplicon	1,597,584	1,143,202	14,984	1,414.55	VIRALNGS	20.56	07/02/13	SAMN08724935
KEN/Kilifi/WGS/1149_07/02/2013	MH181900	GA2	6-amplicon	1,333,010	777,046	14,978	8,232.99	VIRALNGS	20.88	07/02/13	SAMN08724936
KEN/Kilifi/WGS/1151_10/02/2013	MH181989	ON1	6-amplicon	1,727,108	159,996	15,037	1,869.62	VIRALNGS	18.67	10/02/13	SAMN08724937
KEN/Kilifi/WGS/1153_12/03/2013	MH181990	ON1	6-amplicon	1,520,970	887,602	15,001	10,661.08	VIRALNGS	18.79	12/03/13	SAMN08724938
KEN/Kilifi/WGS/1155_13/03/2013	MH181901	GA2	6-amplicon	1,024,466	779,830	14,985	10,443.44	VIRALNGS	24.58	24/05/13	SAMN08724939
KEN/Kilifi/WGS/1159_24/05/2013	MH181902	GA2	6-amplicon	1,795,530	959,456	14,981	11,739.93	VIRALNGS	22.76	24/05/13	SAMN08724940

KEN/Kilifi/WGS/1160_31/05/2013	MH181903	GA2	6-amplicon	1,349,884	520,174	14,764	6,539.51	VIRALNGS	22.57	31/05/13	SAMN08724941
KEN/Kilifi/WGS/1162_12/10/2013	MH181991	ON1	6-amplicon	1,003,770	784,072	15,047	10,930.69	VIRALNGS	21.5	12/10/13	SAMN08724942
KEN/Kilifi/WGS/1163_15/10/2013	MH181992	ON1	6-amplicon	1,071,636	612,044	15,053	8,500.36	VIRALNGS	22.21	15/10/13	SAMN08724943
KEN/Kilifi/WGS/1166_28/10/2013	MH181993	ON1	6-amplicon	845,020	193,328	15,021	2,668.08	VIRALNGS	26.99	28/10/13	SAMN08724944
KEN/Kilifi/WGS/1167_04/11/2013	MH181994	ON1	6-amplicon	1,165,720	474,594	15,023	6,649.96	VIRALNGS	25.14	04/11/13	SAMN08724945
KEN/Kilifi/WGS/1168_07/11/2013	MH181995	ON1	6-amplicon	1,209,356	203,814	14,979	2,880.96	VIRALNGS	23.27	07/11/13	SAMN08724946
KEN/Kilifi/WGS/1169_07/11/2013	MH181904	GA2	6-amplicon	1,606,550	1,029,916	15,011	10,948.78	VIRALNGS	19.27	07/11/13	SAMN08724947
KEN/Kilifi/WGS/1171_12/11/2013	MH181996	ON1	6-amplicon	947,458	479,690	14,828	6,448.30	VIRALNGS	19.7	12/11/13	SAMN08724948
KEN/Kilifi/WGS/1173_19/11/2013	MH181997	ON1	6-amplicon	1,058,828	662,216	15,052	9,348.85	VIRALNGS	30.66	19/11/13	SAMN08724949
KEN/Kilifi/WGS/1582_01/04/2014	MH181998	ON1	6-amplicon	1,568,332	687,440	15,026	8,437.15	VIRALNGS	24.16	13/04/14	SAMN08724950
KEN/Kilifi/WGS/1583_13/04/2014	MH181999	ON1	6-amplicon	302,368	39,148	14,734	510.27	VIRALNGS	26.44	15/04/14	SAMN08724951
KEN/Kilifi/WGS/1215_15/04/2014	MH181905	GA2	6-amplicon	1,729,644	998,364	15,044	11,774.25	VIRALNGS	21.29	15/04/14	SAMN08724952
KEN/Kilifi/WGS/1584_23/04/2014	MH182000	ON1	6-amplicon	1,842,612	244,414	14,868	2,917.30	VIRALNGS	26.07	26/11/14	SAMN08724953
KEN/Kilifi/WGS/1246_26/11/2014	MH182001	ON1	6-amplicon	1,174,582	955,666	15,187	12,678.17	VIRALNGS	21.34	26/11/14	SAMN08724954
KEN/Kilifi/WGS/1247_04/12/2014	MH182002	ON1	6-amplicon	706,526	491,376	15,055	6,409.80	VIRALNGS	24.28	04/12/14	SAMN08724955
KEN/Kilifi/WGS/1249_05/12/2014	MH182003	ON1	6-amplicon	2,218,820	1,646,984	15,057	19,674.11	VIRALNGS	24.08	05/12/14	SAMN08724956
KEN/Kilifi/WGS/1250_06/12/2014	MH182004	ON1	6-amplicon	218,950	110,400	14,825	1,493.04	VIRALNGS	24.93	06/12/14	SAMN08724957
KEN/Kilifi/WGS/1251_06/12/2014	MH182005	ON1	6-amplicon	1,174,208	89,898	14,802	1,320.67	VIRALNGS	26.32	06/12/14	SAMN08724958
KEN/Kilifi/WGS/1252_07/12/2014	MH182006	ON1	6-amplicon	1,114,664	424,006	15,043	5,840.78	VIRALNGS	24.72	07/12/14	SAMN08724959
KEN/Kilifi/WGS/1253_08/12/2014	MH182007	ON1	6-amplicon	1,657,878	791,536	15,047	10,202.00	VIRALNGS	27.31	08/12/14	SAMN08724960
KEN/Kilifi/WGS/1254_08/12/2014	MH182008	ON1	6-amplicon	2,111,452	1,448,258	15,052	19,567.76	VIRALNGS	24.71	08/12/14	SAMN08724961
KEN/Kilifi/WGS/1256_09/12/2014	MH182009	ON1	6-amplicon	929,072	446,432	15,167	5,904.01	VIRALNGS	27.5	09/12/14	SAMN08724962
KEN/Kilifi/WGS/1257_10/12/2014	MH182010	ON1	6-amplicon	905,314	679,084	15,023	9,435.24	VIRALNGS	24.48	10/12/14	SAMN08724963
KEN/Kilifi/WGS/1258_11/12/2014	MH182011	ON1	6-amplicon	1,549,978	1,255,922	15,058	16,074.53	VIRALNGS	21.74	11/12/14	SAMN08724964
KEN/Kilifi/WGS/1260_14/12/2014	MH182012	ON1	6-amplicon	1,050,160	881,444	15,060	11,114.68	VIRALNGS	23.38	14/12/14	SAMN08724965
KEN/Kilifi/WGS/1261_14/12/2014	MH182013	ON1	6-amplicon	1,109,236	931,816	15,047	12,570.43	VIRALNGS	25.45	14/12/14	SAMN08724966
KEN/Kilifi/WGS/1263_15/12/2014	MH182014	ON1	6-amplicon	1,227,836	608,602	15,180	8,407.71	VIRALNGS	26.53	15/12/14	SAMN08724967
KEN/Kilifi/WGS/1264_15/12/2014	MH182015	ON1	6-amplicon	713,002	11,594	14,172	179.84	VIRALNGS	32.25	15/12/14	SAMN08724968
KEN/Kilifi/WGS/1266_17/12/2014	MH182016	ON1	6-amplicon	1,620,164	1,332,364	15,059	16,697.38	VIRALNGS	23.34	17/12/14	SAMN08724969
KEN/Kilifi/WGS/1268_17/12/2014	MH182017	ON1	6-amplicon	2,064,268	1,740,450	15,148	21,533.23	VIRALNGS	22.85	17/12/14	SAMN08724970
KEN/Kilifi/WGS/1269_17/12/2014	MH182018	ON1	6-amplicon	1,193,216	830,040	15,187	11,270.51	VIRALNGS	25.02	17/12/14	SAMN08724971
KEN/Kilifi/WGS/1271_18/12/2014	MH182019	ON1	6-amplicon	966,686	822,958	15,098	11,699.58	VIRALNGS	22.74	18/12/14	SAMN08724972
KEN/Kilifi/WGS/1273_19/12/2014	MH182020	ON1	6-amplicon	1,279,716	942,368	15,053	12,026.06	VIRALNGS	24.03	19/12/14	SAMN08724973
KEN/Kilifi/WGS/1274_21/12/2014	MH182021	ON1	6-amplicon	1,097,012	811,778	15,184	10,575.22	VIRALNGS	26.9	21/12/14	SAMN08724974
KEN/Kilifi/WGS/1275_22/12/2014	MH182022	ON1	6-amplicon	1,650,292	1,292,028	15,047	17,744.98	VIRALNGS	25.17	22/12/14	SAMN08724975
KEN/Kilifi/WGS/1278_25/12/2014	MH182023	ON1	6-amplicon	1,064,228	735,742	15,155	9,942.56	VIRALNGS	28.76	25/12/14	SAMN08724976
KEN/Kilifi/WGS/1279_27/12/2014	MH182024	ON1	6-amplicon	757,922	130,334	14,824	1,797.32	VIRALNGS	27.91	27/12/14	SAMN08724977
KEN/Kilifi/WGS/1281_30/12/2014	MH182025	ON1	6-amplicon	921,080	610,496	15,164	8,271.09	VIRALNGS	22.41	30/12/14	SAMN08724978
KEN/Kilifi/WGS/1282_31/12/2014	MH182026	ON1	6-amplicon	1,648,622	676,458	15,020	8,667.22	VIRALNGS	29.32	31/12/14	SAMN08724979
KEN/Kilifi/WGS/1283_31/12/2014	MH182027	ON1	6-amplicon	1,315,628	694,854	15,031	9,598.81	VIRALNGS	24.97	31/12/14	SAMN08724980
KEN/Kilifi/WGS/1284_02/01/2015	MH182028	ON1	6-amplicon	1,059,850	813,254	15,184	10,727.02	VIRALNGS	24.28	02/01/15	SAMN08724981
KEN/Kilifi/WGS/1286_04/01/2015	MH182029	ON1	6-amplicon	2,048,806	1,502,422	15,056	18,679.43	VIRALNGS	25.14	04/01/15	SAMN08724982
KEN/Kilifi/WGS/1287_06/01/2015	MH182030	ON1	6-amplicon	1,320,088	1,006,680	15,204	13,479.43	VIRALNGS	22.91	06/01/15	SAMN08724983
KEN/Kilifi/WGS/1288_07/01/2015	MH182031	ON1	6-amplicon	537,376	426,692	15,059	5,429.30	VIRALNGS	23.91	07/01/15	SAMN08724984
KEN/Kilifi/WGS/1289_08/01/2015	MH182032	ON1	6-amplicon	1,099,200	783,598	15,044	11,047.58	VIRALNGS	23.81	08/01/15	SAMN08724985
KEN/Kilifi/WGS/1290_08/01/2015	MH182033	ON1	6-amplicon	930,504	107,914	14,796	1,529.49	VIRALNGS	34.87	08/01/15	SAMN08724986
KEN/Kilifi/WGS/1292_09/01/2015	MH182034	ON1	6-amplicon	770,526	7,412	14,216	111.73	VIRALNGS	28.13	09/01/15	SAMN08724987
KEN/Kilifi/WGS/1293_24/01/2015	MH182035	ON1	6-amplicon	1,040,044	848,258	15,056	10,945.92	VIRALNGS	24.24	24/01/15	SAMN08724988
KEN/Kilifi/WGS/1295_27/01/2015	MH182036	ON1	6-amplicon	959,732	725,710	15,206	9,327.55	VIRALNGS	23.86	27/01/15	SAMN08724989
KEN/Kilifi/WGS/1296_29/01/2015	MH182037	ON1	6-amplicon	1,024,190	681,324	15,052	9,406.83	VIRALNGS	25.01	29/01/15	SAMN08724990
KEN/Kilifi/WGS/1298_31/01/2015	MH182038	ON1	6-amplicon	765,634	523,320	15,047	7,236.79	VIRALNGS	24.66	31/01/15	SAMN08724991
KEN/Kilifi/WGS/1299_04/02/2015	MH182039	ON1	6-amplicon	1,390,092	34,784	14,741	497.77	VIRALNGS	31.57	04/02/15	SAMN08724992
KEN/Kilifi/WGS/1301_08/02/2015	MH182040	ON1	6-amplicon	1,184,664	633,118	15,117	8,281.10	VIRALNGS	25.06	08/02/15	SAMN08724993
KEN/Kilifi/WGS/1302_13/02/2015	MH182041	ON1	6-amplicon	1,134,242	469,232	14,848	6,827.43	VIRALNGS	25.69	13/02/15	SAMN08724994
KEN/Kilifi/WGS/1305_21/02/2015	MH182042	ON1	6-amplicon	912,242	614,448	15,102	8,643.56	VIRALNGS	28.16	21/02/15	SAMN08724995
KEN/Kilifi/WGS/1307_26/02/2015	MH182043	ON1	6-amplicon	1,032,236	867,040	15,033	11,866.93	VIRALNGS	24.37	26/02/15	SAMN08724996
KEN/Kilifi/WGS/1308_05/03/2015	MH182044	ON1	6-amplicon	1,204,456	1,053,810	15,201	13,377.58	VIRALNGS	24.26	05/03/15	SAMN08724997
KEN/Kilifi/WGS/1309_08/03/2015	MH182045	ON1	6-amplicon	1,110,724	915,046	15,053	12,126.92	VIRALNGS	24.14	08/03/15	SAMN08724998
KEN/Kilifi/WGS/1311_21/03/2015	MH182046	ON1	6-amplicon	307,420	193,146	14,740	2,480.30	VIRALNGS	27.9	21/03/15	SAMN08724999
KEN/Kilifi/WGS/1312_22/03/2015	MH182047	ON1	6-amplicon	1,722,762	1,296,644	15,050	15,953.55	VIRALNGS	24.91	22/03/15	SAMN08725000
KEN/Kilifi/WGS/1313_27/03/2015	MH182048	ON1	6-amplicon	1,143,280	429,554	14,827	5,977.84	VIRALNGS	27.51	27/03/15	SAMN08725001
KEN/Kilifi/WGS/1314_31/03/2015	MH181906	GA2	6-amplicon	1,625,342	1,010,186	15,022	11,311.20	VIRALNGS	20.75	31/03/15	SAMN08725002
KEN/Kilifi/WGS/1315_01/04/2015	MH182049	ON1	6-amplicon	1,107,120	740,496	15,066	9,340.28	VIRALNGS	23.95	01/04/15	SAMN08725003
KEN/Kilifi/WGS/1318_08/04/2015	MH182050	ON1	6-amplicon	900,852	759,034	15,202	9,913.31	VIRALNGS	23.52	08/04/15	SAMN08725004
KEN/Kilifi/WGS/1320_12/04/2015	MH182051	ON1	6-amplicon	1,698,698	1,185,294	15,048	14,503.36	VIRALNGS	24.15	12/04/15	SAMN08725005
KEN/Kilifi/WGS/1321_17/04/2015	MH182052	ON1	6-amplicon	1,074,506	944,630	15,019	12,360.20	VIRALNGS	25.73	17/04/15	SAMN08725006
KEN/Kilifi/WGS/1322_17/04/2014	MH181907	GA2	6-amplicon	1,398,136	367,740	14,757	4,605.53	VIRALNGS	23.73	17/04/14	SAMN08725007
KEN/Kilifi/WGS/1297_29/01/2015	MH182053	ON1	6-amplicon	1,598,292	1,046	13,966	39.95	VIRALNGS	NA	29/01/15	SAMN08725008
KEN/Kilifi/WGS/1585_17/02/2016	MH182054	ON1	6-amplicon	1,149,120	700,486	15,082	8,828.96	VIRALNGS	22.09	17/02/16	SAMN08725009
KEN/Kilifi/WGS/1586_28/01/2016	MH182055	ON1	6-amplicon	1,156,030	266,436	14,704	3,938.20	VIRALNGS	22.58	28/01/16	SAMN08725010
KEN/Kilifi/WGS/1587_08/02/2016	MH182056	ON1	6-amplicon	939,876	48,486	14,882	712.62	VIRALNGS	25.82	08/02/16	SAMN08725011
KEN/Kilifi/WGS/1588_29/03/2016	MH182057	ON1	6-amplicon	1,021,102	391,966	14,993	4,937.68	VIRALNGS	25.32	29/03/16	SAMN08725012
KEN/Kilifi/WGS/1589_26/01/2016	MH182058	ON1	6-amplicon	1,281,062	99,496	14,660	1,353.70	VIRALNGS	25.92	26/01/16	SAMN08725013
KEN/Kilifi/WGS/1590_01/02/2016	MH182059	ON1	6-amplicon	1,153,340	441,172	15,035	5,304.08	VIRALNGS	22.02	01/02/16	SAMN08725014
KEN/Kilifi/WGS/1591_18/01/2016	MH182060	ON1	6-amplicon	1,124,140	191,242	15,026	2,565.53	VIRALNGS	22.5	18/01/16	SAMN08725015
KEN/Kilifi/WGS/1592_07/04/2016	MH182061	ON1	6-amplicon	1,089,038	647,740	15,078	8,421.34	VIRALNGS	27.7	07/04/16	SAMN08725016
