# Supplementary material for: Whole genome analysis of local Kenyan and global sequences unravels the epidemiological and molecular evolutionary dynamics of RSV genotype ON1 strains
Source: Virus Evol. 2018 Sep 24;4(2):vey027. doi: 10.1093/ve/vey027 (PMC6153471; doi:10.1093/ve/vey027)
Supplement: Supplementary S3 Table [file vey027_supplementary_s3_table.pdf]

Genome Position	CDS	CDS Nt Pos.	CDS AA Pos.	CDS Codon Pos.	Nt Change	Variant Frequency	Coverage	Polymorphism	AA Change
2					-AATTTGA	1.0% -> 1.3%	75 -> 96	Deletion	
9					T -> C/-	7.3%/1.0%	96	Mixture	
10					-AA	1.00%	97 -> 99	Deletion	
80	NS1	37	13	1	T -> C	1.50%	137	transition	
112	NS1	69	23	3	A -> G	2.60%	151	transition	
134	NS1	91	31	1	A -> G	2.70%	149	transition	T -> A
172	NS1	129	43	3	G -> A	6.30%	144	transition	
175	NS1	132	44	3	A -> T	3.50%	143	transversion	
187	NS1	144	48	3	A -> G	1.40%	142	transition	
196	NS1	153	51	3	G -> A	1.40%	139	transition	
244	NS1	201	67	3	T -> C	1.70%	172	transition	
359	NS1	316	106	1	G -> T	1.10%	178	transversion	D -> Y
460	NS1	417	139	3	A -> G	1.70%	177	transition	
498					C -> A	2.20%	178	transversion	
507					T -> C	2.80%	179	transition	
518					+AAA	1.70%	181	Insertion	
531					A -> G	3.40%	177	transition	
532					G -> A	4.00%	177	transition	
534					T -> C	5.10%	177	transition	
561					A -> G	99.40%	177	transition	
567					C -> T	1.70%	176	transition	
571					C -> T	1.10%	177	transition	
605	NS2	33	11	3	A -> G	2.30%	176	transition	
629	NS2	57	19	3	A -> G	1.70%	176	transition	
638	NS2	66	22	3	A -> G	82.40%	176	transition	
641	NS2	69	23	3	T -> G	5.10%	176	transversion	
647	NS2	75	25	3	T -> C	2.30%	177	transition	
660	NS2	88	30	1	T -> C	88.80%	178	transition	
734	NS2	162	54	3	A -> G	1.10%	179	transition	
752	NS2	180	60	3	A -> G	2.20%	179	transition	
761	NS2	189	63	3	C -> T	1.10%	179	transition	
770	NS2	198	66	3	A -> G	1.10%	180	transition	
780	NS2	208	70	1	T -> C	1.10%	180	transition	
788	NS2	216	72	3	A -> G	1.10%	180	transition	
800	NS2	228	76	3	T -> C	1.10%	180	transition	
863	NS2	291	97	3	C -> T	3.30%	181	transition	
879	NS2	307	103	1	T -> C	6.60%	182	transition	
896	NS2	324	108	3	T -> C	1.10%	182	transition	
902	NS2	330	110	3	T -> C	1.10%	182	transition	
956					A -> C/T	47.0%/36.5%	181	SNP	
978					T -> C	5.60%	180	transition	
981					C -> T	1.10%	180	transition	
983					A -> G	81.10%	180	transition	
985					T -> C	1.10%	180	transition	
986					T -> C	1.70%	180	transition	
993					T -> C	1.10%	180	transition	
1008					G -> A	1.10%	182	transition	
1012					A -> G	1.10%	182	transition	
1017					G -> A	4.40%	182	transition	
1024					A -> G	1.10%	181	transition	
1064					A -> G	1.60%	183	transition	
1189	N	105	35	3	T -> A	36.50%	181	transversion	
1204	N	120	40	3	G -> T	6.60%	182	transversion	
1231	N	147	49	3	C -> T	36.50%	181	transition	
1235	N	151	51	1	T -> C	28.20%	181	transition	
1255	N	171	57	3	T -> C	2.80%	181	transition	
1267	N	183	61	3	C -> T	37.40%	182	transition	
1286	N	202	68	1	T -> C	4.40%	182	transition	
1300	N	216	72	3	T -> C	1.10%	182	transition	
1342	N	258	86	3	G -> A	1.10%	181	transition	
1495	N	411	137	3	A -> G	1.10%	182	transition	
1501	N	417	139	3	A -> G	1.10%	182	transition	
1522	N	438	146	3	T -> C	1.10%	182	transition	
1543	N	459	153	3	T -> C	9.90%	182	transition	
1615	N	531	177	3	T -> A	88.50%	183	transversion	
1630	N	546	182	3	G -> A	1.10%	183	transition	
1642	N	558	186	3	T -> C	3.30%	183	transition	
1645	N	561	187	3	T -> C	2.20%	183	transition	
1675	N	591	197	3	T -> C	1.10%	183	transition	
1699	N	615	205	3	A -> C	1.60%	183	transversion	
1720	N	636	212	3	G -> A	1.10%	183	transition	
1765	N	681	227	3	T -> C	3.30%	183	transition	
1801	N	717	239	3	T -> C	2.20%	183	transition	
1807	N	723	241	3	G -> A	6.60%	183	transition	
1813	N	729	243	3	T -> C	1.10%	183	transition	
1820	N	736	246	1	T -> C	2.20%	183	transition	
1825	N	741	247	3	T -> C	1.10%	183	transition	
1827	N	743	248	2	T -> A	3.30%	183	transversion	M -> K
1859	N	775	259	1	C -> A	2.70%	184	transversion	
1861	N	777	259	3	G -> A	1.60%	184	transition	
1876	N	792	264	3	A -> G	1.60%	184	transition	
1885	N	801	267	3	T -> A	1.10%	184	transversion	
1885	N	801	267	3	T -> C	1.10%	184	transition	
1888	N	804	268	3	A -> G	4.90%	184	transition	
1921	N	837	279	3	A -> T	6.50%	184	transversion	
2032	N	948	316	3	A -> G	1.60%	184	transition	

2062	N	978	326	3	C -> T	1.10%	184	transition	
2080	N	996	332	3	C -> T	1.10%	184	transition	
2146	N	1062	354	3	T -> C	1.10%	184	transition	
2170	N	1086	362	3	G -> A	2.20%	184	transition	
2191	N	1107	369	3	C -> T	83.70%	184	transition	
2200	N	1116	372	3	A -> G	3.80%	184	transition	
2267					(A)6 -> (A)7	4.90%	184	Insertion (tandem repeat)	
2267					(A)6 -> (A)5	2.70%	183	Deletion (tandem repeat)	
2272					A -> G	77.00%	183	transition	
2273					G -> A	8.20%	183	transition	
2366	P	75	25	3	G -> A	1.10%	182	transition	
2478	P	187	63	1	A -> G	37.70%	183	transition	I -> V
2495	P	204	68	3	G -> A	5.40%	184	transition	
2513	P	222	74	3	G -> A	1.10%	184	transition	
2522	P	231	77	3	T -> C	98.90%	184	transition	
2525	P	234	78	3	T -> C	3.30%	184	transition	
2585	P	294	98	3	C -> T	98.40%	184	transition	
2588	P	297	99	3	A -> T	5.40%	184	transversion	
2600	P	309	103	3	A -> G	2.70%	184	transition	
2663	P	372	124	3	T -> C	1.10%	184	transition	
2678	P	387	129	3	T -> C	1.60%	184	transition	
2753	P	462	154	3	A -> G	1.10%	184	transition	
2756	P	465	155	3	G -> A	8.70%	184	transition	
2768	P	477	159	3	T -> C	83.70%	184	transition	
2780	P	489	163	3	G -> A	2.20%	184	transition	
2804	P	513	171	3	T -> C	3.80%	184	transition	
2837	P	546	182	3	G -> A	88.00%	184	transition	
2858	P	567	189	3	T -> C	2.20%	184	transition	
2873	P	582	194	3	T -> C	3.30%	184	transition	
2966	P	675	225	3	C -> T	2.20%	184	transition	
3011	P	720	240	3	T -> C	1.60%	184	transition	
3019					T -> C	1.60%	184	transition	
3054					T -> C	1.10%	184	transition	
3066					A -> G	1.10%	184	transition	
3085					T -> C	4.90%	184	transition	
3086					C -> T	2.20%	184	transition	
3091					C -> T	2.70%	184	transition	
3099					T -> C	5.40%	184	transition	
3103					C -> T	99.50%	184	transition	
3106					C -> T	88.00%	184	transition	
3109					C -> T	85.90%	184	transition	
3110					T -> C	2.20%	184	transition	
3123					A -> T	1.10%	184	transversion	
3135					G -> T/A	1.6%/1.1%	184	SNP	
3136					C -> T	2.20%	184	transition	
3142					T -> C	7.10%	184	transition	
3143					A -> T	1.60%	184	transversion	
3159					(A)6 -> (A)7	1.10%	184	Insertion (tandem repeat)	
3159					(A)6 -> (A)5	1.10%	184	Deletion (tandem repeat)	
3171					T -> C	99.50%	184	transition	
3181					(A)7 -> (A)8	1.60%	184	Insertion (tandem repeat)	
3217	M	18	6	3	C -> T	85.20%	183	transition	
3229	M	30	10	3	G -> A	8.70%	183	transition	
3247	M	48	16	3	T -> A	1.60%	183	transversion	
3266	M	67	23	1	C -> T	4.40%	183	transition	
3277	M	78	26	3	C -> T	1.10%	183	transition	
3310	M	111	37	3	C -> T	2.20%	183	transition	
3322	M	123	41	3	A -> G	2.20%	183	transition	
3339	M	140	47	2	T -> A	37.70%	183	transversion	L -> Q
3394	M	195	65	3	C -> T	1.10%	183	transition	
3397	M	198	66	3	A -> G	88.50%	183	transition	
3403	M	204	68	3	T -> C	88.50%	183	transition	
3409	M	210	70	3	G -> A	99.50%	183	transition	
3418	M	219	73	3	G -> A	1.60%	183	transition	M -> I
3466	M	267	89	3	C -> T	1.10%	183	transition	
3475	M	276	92	3	C -> T	33.90%	183	transition	
3505	M	306	102	3	G -> A	2.20%	183	transition	
3538	M	339	113	3	G -> A	1.10%	182	transition	
3548	M	349	117	1	C -> T	1.60%	182	transition	
3565	M	366	122	3	A -> T	4.40%	182	transversion	
3643	M	444	148	3	A -> G	1.60%	182	transition	
3670	M	471	157	3	A -> G	4.90%	182	transition	
3674	M	475	159	1	A -> G	1.10%	182	transition	I -> V
3697	M	498	166	3	T -> C	1.10%	183	transition	
3703	M	504	168	3	T -> C	88.50%	183	transition	
3721	M	522	174	3	G -> A	9.30%	183	transition	
3745	M	546	182	3	C -> T	1.10%	183	transition	
3763	M	564	188	3	A -> C	97.30%	182	transversion	
3763	M	564	188	3	A -> T	2.20%	182	transversion	
3787	M	588	196	3	T -> C	1.60%	182	transition	
3800	M	601	201	1	C -> T	1.10%	182	transition	
3823	M	624	208	3	C -> T	4.90%	182	transition	
3844	M	645	215	3	C -> T	1.10%	183	transition	
3969	M	770	257	2	A -> G	38.30%	183	transition	
3972					C -> T	4.40%	183	transition	
3976					T -> C	1.60%	183	transition	
3977					T -> C/-	2.2%/1.6%	183	Mixture	

3986					T -> A	10.40%	183	transversion	
3994					T -> C	82.00%	183	transition	
3998					T -> C	2.20%	183	transition	
4002					T -> C	82.00%	183	transition	
4009					T -> C	4.90%	183	transition	
4010					T -> A	1.10%	183	transversion	
4013					T -> C	82.50%	183	transition	
4014					A -> T	4.90%	183	transversion	
4017					T -> C	82.00%	183	transition	
4021					T -> C	2.20%	183	transition	
4030					T -> C	82.00%	183	transition	
4031					C -> T	1.10%	183	transition	
4039					T -> C	81.90%	182	transition	
4049					C -> T	6.00%	182	transition	
4053					C -> T	7.10%	182	transition	
4054					T -> C	81.90%	182	transition	
4057					G -> T	1.10%	182	transversion	
4064					T -> C	81.90%	182	transition	
4069					C -> T	11.50%	182	transition	
4093					A -> T	2.70%	182	transversion	
4107					C -> T	2.20%	182	transition	
4112					T -> A	1.60%	182	transversion	
4117					C -> T	3.80%	182	transition	
4118					G -> A	99.50%	182	transition	
4119					G -> A	4.40%	182	transition	
4123					C -> A	5.50%	182	transversion	
4129					T -> A	1.10%	182	transversion	
4131					A -> G	1.10%	182	transition	
4143					(A)6 -> (A)7	1.10%	184	Insertion (tandem repeat)	
4143					(A)6 -> (A)5	2.20%	182	Deletion (tandem repeat)	
4148					-AT	3.30%	182	Deletion	
4172					G -> A	1.10%	181	transition	
4175					G -> A	1.10%	181	transition	
4186					T -> C	1.70%	181	transition	
4189					T -> C	1.10%	181	transition	
4195					C -> G/A	36.5%/1.1%	181	SNP	
4228					T -> C	1.60%	182	transition	
4238					C -> T	1.10%	182	transition	
4287	SH	48	16	3	T -> C	1.10%	182	transition	
4296	SH	57	19	3	A -> G	1.10%	181	transition	
4297	SH	58	20	1	T -> C	99.50%	182	transition	
4300	SH	61	21	1	A -> G	1.10%	181	transition	I -> V
4300	SH	61	21	1	A -> T	1.10%	181	transversion	I -> L
4303	SH	64	22	1	C -> T	35.70%	182	transition	H -> Y
4335	SH	96	32	3	A -> C	99.40%	181	transversion	
4365	SH	126	42	3	C -> T	1.10%	181	transition	
4374	SH	135	45	3	C -> T	1.10%	181	transition	
4385	SH	146	49	2	T -> C	1.10%	181	transition	V -> A
4386	SH	147	49	3	A -> G	1.10%	181	transition	
4391	SH	152	51	2	A -> T	2.80%	181	transversion	H -> L
4397	SH	158	53	2	A -> G	2.80%	181	transition	K -> R
4398	SH	159	53	3	A -> G	1.10%	181	transition	
4423	SH	184	62	1	G -> A	5.50%	181	transition	V -> I
4459					A -> G	1.10%	182	transition	
4463					T -> C	3.30%	182	transition	
4480					G -> A	87.90%	182	transition	
4482					G -> A	1.60%	182	transition	
4495					C -> T	84.20%	183	transition	
4496					T -> C	3.30%	183	transition	
4520					T -> C	3.30%	183	transition	
4521					T -> C	99.50%	183	transition	
4532					C -> T	1.10%	183	transition	
4540					T -> C	3.30%	182	transition	
4543					A -> G	1.10%	182	transition	
4558					A -> T	3.30%	182	transversion	
4561					(A)5 -> (A)6	3.80%	184	Insertion (tandem repeat)	
4561					(A)5 -> (A)6	57.40%	183	Insertion (tandem repeat)	
4570					C -> T	1.10%	182	transition	
4575					C -> T	4.40%	182	transition	
4579					G -> A	1.10%	182	transition	
4585					G -> A	38.50%	182	transition	
4592					A -> G	2.20%	183	transition	
4598					(A)5 -> (A)6	4.90%	183	Insertion (tandem repeat)	
4606					C -> T	4.40%	183	transition	
4647	G	23	8	2	G -> A	1.60%	183	transition	R -> H
4651	G	27	9	3	C -> T	1.10%	183	transition	
4684	G	60	20	3	C -> T	2.20%	183	transition	
4691	G	67	23	1	CTA -> TTG	9.80%	183	Substitution	
4693	G	69	23	3	A -> G	71.60%	183	transition	
4708	G	84	28	3	G -> A	4.90%	183	transition	
4756	G	132	44	3	T -> C	1.10%	184	transition	
4763	G	139	47	1	G -> A	3.30%	184	transition	A -> T
4793	G	169	57	1	G -> A	32.20%	183	transition	A -> T
4842	G	218	73	2	C -> A	3.30%	183	transversion	T -> N
4864	G	240	80	3	G -> A	1.60%	183	transition	
4867	G	243	81	3	C -> T	1.10%	183	transition	
4885	G	261	87	3	C -> T	26.10%	184	transition	

4900	G	276	92	3	C -> T	2.70%	184	transition	
4907	G	283	95	1	C -> T	1.60%	183	transition	P -> S
4909	G	285	95	3	C -> T	1.10%	183	transition	
4927	G	303	101	3	C -> A	1.10%	183	transversion	F -> L
4945	G	321	107	3	T -> C	5.50%	183	transition	
4968	G	344	115	2	T -> C	6.60%	183	transition	L -> P
4969	G	345	115	3	A -> T	1.10%	183	transversion	
4977	G	353	118	2	C -> T	4.90%	183	transition	T -> I
4993	G	369	123	3	G -> A	1.10%	183	transition	
5007	G	383	128	2	C -> T	2.20%	182	transition	S -> F
5021	G	397	133	1	A -> G	1.10%	182	transition	I -> V
5031	G	407	136	2	C -> T	30.80%	182	transition	T -> I
5046	G	422	141	2	T -> C	1.10%	182	transition	I -> T
5048	G	424	142	1	CA -> TT	87.90%	182	Substitution	Q -> L
5053	G	429	143	3	T -> C	6.60%	182	transition	
5076	G	452	151	2	G -> A	1.10%	183	transition	R -> H
5081	G	457	153	1	A -> G	1.60%	183	transition	N -> D
5083	G	459	153	3	T -> C	1.10%	183	transition	
5095	G	471	157	3	C -> T	37.70%	183	transition	
5096	G	472	158	1	A -> C	1.60%	183	transversion	K -> Q
5104	G	480	160	3	C -> T	1.10%	183	transition	
5158	G	534	178	3	T -> C	97.30%	182	transition	
5221	G	597	199	3	C -> T	2.20%	182	transition	
5226	G	602	201	2	G -> A	98.90%	182	transition	R -> K
5230	G	606	202	3	C -> T	31.90%	182	transition	
5241	G	617	206	2	C -> A	37.40%	182	transversion	P -> Q
5244	G	620	207	2	C -> T	3.30%	182	transition	T -> I
5246	G	622	208	1	A -> C	88.50%	182	transversion	I -> L
5275	G	651	217	3	T -> C	4.90%	182	transition	
5277	G	653	218	2	A -> G	2.20%	182	transition	Q -> R
5297	G	673	225	1	G -> A	1.10%	182	transition	V -> I
5302	G	678	226	3	C -> A	1.60%	183	transversion	
5305	G	681	227	3	C -> T	79.80%	183	transition	
5308	G	684	228	3	C -> T	1.60%	183	transition	
5309	G	685	229	1	A -> G	1.10%	183	transition	K -> E
5314	G	690	230	3	C -> T	83.60%	183	transition	
5319	G	695	232	2	A -> G	84.20%	183	transition	E -> G
5333	G	709	237	1	G -> A	88.50%	183	transition	D -> N
5335	G	711	237	3	C -> T	1.10%	183	transition	
5338	G	714	238	3	C -> T	1.60%	183	transition	
5340	G	716	239	2	CC -> TT	1.10%	183	Substitution	T -> I
5341	G	717	239	3	C -> T	73.20%	183	transition	
5346	G	722	241	2	C -> T	1.10%	183	transition	T -> I
5355	G	731	244	2	G -> A	3.80%	183	transition	R -> K
5361	G	737	246	2	C -> T	1.10%	183	transition	T -> I
5363	G	739	247	1	C -> T	4.40%	183	transition	
5372	G	748	250	1	T -> C	5.50%	183	transition	S -> P
5373	G	749	250	2	C -> T	1.10%	183	transition	S -> F
5377	G	753	251	3	C -> A	1.60%	183	transversion	N -> K
5382	G	758	253	2	C -> A	84.20%	183	transversion	T -> K
5390	G	766	256	1	C -> T	1.10%	183	transition	P -> S
5396	G	772	258	1	C -> T	4.40%	183	transition	H -> Y
5411	G	787	263	1	G -> A	1.60%	183	transition	E -> K
5424	G	800	267	2	C -> T	2.70%	183	transition	S -> L
5435	G	811	271	1	G -> A	1.10%	183	transition	E -> K
5441	G	817	273	1	A -> C	7.10%	183	transversion	N -> H
5441	G	817	273	1	A -> T	81.40%	183	transversion	N -> Y
5444	G	820	274	1	CT -> TC	7.10%	183	Substitution	L -> S
5445	G	821	274	2	T -> C	77.00%	183	transition	L -> P
5462	G	838	280	1	C -> T	17.50%	183	transition	H -> Y
5462	G	838	280	1	CAT -> TAC	81.40%	183	Substitution	H -> Y
5472	G	848	283	2	C -> T	1.10%	183	transition	S -> F
5475	G	851	284	2	+GTCAAGAGGAAACCCTCCACTCAACCACCCCCGAAGGCCATCCAAGCCCATCACAAGTCCATACAACATCCG	2.20%	184	Insertion	E -> GQEETLHSTTPEGHPSPSQVHTTSE
5475	G	851	284	2	+GTCAAGAGGAAACCCTCCACTCAACCACCTCCGAAGGCTATCCAAGCCCATCACAAGTCCATACAACATCCG	76.60%	184	Insertion	E -> GQEETLHSTTSEGYPSPSQVHTTSE
5475	G	851	284	2	+GTCAAGAGGAAACCCTCCACTCAACCACCTCCGAAGGCTATCTAAGCCCATCACAAGTCTATACAACATCCG	2.20%	184	Insertion	E -> GQEETLHSTTSEGYLSPSQVYTTSE
5475	G	851	284	2	+TCCATCACAAGTCCATACAACATCCG	1.10%	184	Insertion	
5481	G	857	286	2	C -> T	53.00%	183	transition	P -> L
5493	G	869	290	2	C -> T	82.50%	183	transition	P -> L
5493	G	869	290	2	CA -> TG	1.60%	183	Substitution	P -> L
5501	G	877	293	1	T -> A	1.10%	183	transversion	S -> T
5503	G	879	293	3	C -> A	6.00%	183	transversion	
5506	G	882	294	3	C -> T	1.10%	183	transition	
5509	G	885	295	3	A -> G	30.60%	183	transition	
5510	G	886	296	1	A -> C	3.80%	183	transversion	T -> P

5515	G	891	297	3	A -> C	3.30%	183	transversion	K -> N
5533	G	909	303	3	C -> T	4.40%	183	transition	
5543	G	919	307	1	C -> T	1.10%	183	transition	
5549	G	925	309	1	G -> A	1.10%	183	transition	A -> T
5551	G	927	309	3	C -> T	4.40%	183	transition	
5568	G	944	315	2	G -> A	1.10%	183	transition	R -> K
5572	G	948	316	3	C -> T	1.10%	183	transition	
5577	G	953	318	2	T -> C	3.80%	183	transition	I -> T
5583	G	959	320	2	C -> T	1.10%	183	transition	S -> F
5615	F	18	6	3	C -> T	7.20%	181	transition	
5633	F	36	12	3	C -> A	4.40%	181	transversion	
5633	F	36	12	3	C -> T	5.00%	181	transition	
5649	F	52	18	1	G -> A	1.10%	181	transition	V -> I
5663	F	66	22	3	C -> T	2.80%	181	transition	
5678	F	81	27	3	C -> T	1.70%	181	transition	
5720	F	123	41	3	C -> T	32.60%	181	transition	
5729	F	132	44	3	T -> C	1.60%	182	transition	
5783	F	186	62	3	T -> C	1.10%	180	transition	
5786	F	189	63	3	T -> C	4.40%	180	transition	
5816	F	219	73	3	C -> T	5.00%	181	transition	
5817	F	220	74	1	G -> A	5.50%	181	transition	A -> T
5819	F	222	74	3	T -> C	1.70%	181	transition	
5849	F	252	84	3	T -> C	1.70%	181	transition	
5857	F	260	87	2	A -> G	1.70%	181	transition	K -> R
5902	F	305	102	2	C -> T	1.70%	181	transition	A -> V
5906	F	309	103	3	C -> T	2.20%	181	transition	
5916	F	319	107	1	G -> A	1.10%	181	transition	A -> T
5928	F	331	111	1	C -> T	37.00%	181	transition	
5943	F	346	116	1	G -> A	88.50%	182	transition	D -> N
5961	F	364	122	1	A -> G	81.30%	182	transition	T -> A
5963	F	366	122	3	C -> T	1.60%	182	transition	
5983	F	386	129	2	T -> C	2.70%	182	transition	L -> S
5987	F	390	130	3	C -> T	88.50%	182	transition	
6041	F	444	148	3	C -> T	1.10%	182	transition	
6044	F	447	149	3	C -> T	5.50%	182	transition	
6050	F	453	151	3	C -> A	3.30%	181	transversion	
6059	F	462	154	3	A -> G	2.20%	182	transition	
6069	F	472	158	1	C -> T	1.10%	182	transition	
6074	F	477	159	3	C -> T	1.60%	182	transition	
6104	F	507	169	3	T -> C	1.10%	180	transition	
6111	F	514	172	1	C -> T	7.80%	180	transition	
6113	F	516	172	3	A -> G	2.20%	181	transition	
6116	F	519	173	3	C -> T	3.30%	181	transition	
6143	F	546	182	3	T -> C	5.00%	181	transition	
6170	F	573	191	3	A -> G	84.00%	181	transition	
6185	F	588	196	3	A -> G	1.70%	181	transition	
6213	F	616	206	1	A -> G	1.10%	181	transition	I -> V
6266	F	669	223	3	C -> T	1.10%	181	transition	
6275	F	678	226	3	G -> A	1.10%	181	transition	
6278	F	681	227	3	C -> T	1.10%	181	transition	
6287	F	690	230	3	A -> G	1.10%	182	transition	
6362	F	765	255	3	T -> C	1.10%	181	transition	
6374	F	777	259	3	A -> G	4.40%	181	transition	
6431	F	834	278	3	T -> C	1.10%	182	transition	
6476	F	879	293	3	A -> G	2.20%	182	transition	
6510	F	913	305	1	T -> C	99.50%	183	transition	
6530	F	933	311	3	T -> C	1.10%	182	transition	
6557	F	960	320	3	T -> C	5.00%	181	transition	
6578	F	981	327	3	G -> A	2.20%	182	transition	
6593	F	996	332	3	C -> T	1.10%	182	transition	
6641	F	1044	348	3	A -> T	6.60%	182	transversion	
6677	F	1080	360	3	T -> C	1.60%	182	transition	
6689	F	1092	364	3	G -> A	2.20%	182	transition	
6692	F	1095	365	3	A -> G	83.50%	182	transition	
6731	F	1134	378	3	G -> A	2.20%	182	transition	
6743	F	1146	382	3	T -> C	88.50%	182	transition	
6764	F	1167	389	3	C -> T	1.10%	184	transition	
6770	F	1173	391	3	T -> C	2.70%	184	transition	
6782	F	1185	395	3	T -> C	2.70%	184	transition	
6812	F	1215	405	3	C -> A	1.10%	183	transversion	
6812	F	1215	405	3	C -> T	2.20%	183	transition	
6825	F	1228	410	1	C -> T	2.20%	184	transition	
6830	F	1233	411	3	G -> A	99.50%	184	transition	
6845	F	1248	416	3	C -> T	3.80%	184	transition	
6851	F	1254	418	3	C -> A	1.10%	183	transversion	
6863	F	1266	422	3	T -> C	4.40%	183	transition	
6875	F	1278	426	3	T -> C	3.30%	183	transition	
6941	F	1344	448	3	T -> C	8.80%	182	transition	
6968	F	1371	457	3	T -> C	3.90%	181	transition	
6977	F	1380	460	3	T -> C	4.40%	181	transition	
7031	F	1434	478	3	T -> C	1.70%	181	transition	
7046	F	1449	483	3	C -> T	1.10%	180	transition	
7104	F	1507	503	1	C -> A	2.20%	181	transversion	L -> I
7109	F	1512	504	3	A -> G	32.00%	181	transition	
7181	F	1584	528	3	T -> C	2.80%	181	transition	
7184	F	1587	529	3	T -> C	89.00%	181	transition	
7199	F	1602	534	3	T -> A	5.50%	181	transversion	

7199	F	1602	534	3	T -> C	2.20%	181	transition	
7226	F	1629	543	3	T -> A	89.00%	181	transversion	
7229	F	1632	544	3	A -> T	89.00%	181	transversion	
7253	F	1656	552	3	C -> T	1.10%	181	transition	
7262	F	1665	555	3	A -> G	1.10%	181	transition	
7268	F	1671	557	3	C -> T	1.10%	181	transition	
7277	F	1680	560	3	T -> C	3.30%	181	transition	
7295	F	1698	566	3	T -> C	1.10%	181	transition	
7301	F	1704	568	3	T -> C	4.40%	181	transition	
7313	F	1716	572	3	T -> C	5.00%	181	transition	
7337					T -> C	7.70%	181	transition	
7340					T -> C	2.20%	181	transition	
7359					C -> T	1.10%	181	transition	
7360					A -> G	87.30%	181	transition	
7365					T -> A	4.40%	181	transversion	
7367					A -> G	98.90%	181	transition	
7374					G -> A	32.60%	181	transition	
7392					G -> A	84.50%	181	transition	
7393					G -> A	2.20%	181	transition	
7400					C -> T	3.90%	181	transition	
7405					A -> G	4.40%	181	transition	
7421					A -> T	1.10%	181	transversion	
7423					C -> T	1.10%	181	transition	
7437					C -> T	1.10%	182	transition	
7438					C -> T	3.80%	182	transition	
7453					A -> T	89.00%	181	transversion	
7457					A -> G	1.10%	181	transition	
7461					A -> G	82.20%	180	transition	
7470					T -> A	3.90%	180	transversion	
7484					-AA	87.40%	183	Deletion	
7489					C -> A	95.00%	180	transversion	
7491					A -> G	1.70%	180	transition	
7500					C -> T	6.70%	179	transition	
7569	M2-1	27	9	3	C -> T	2.20%	179	transition	
7611	M2-1	69	23	3	T -> C	3.30%	180	transition	
7650	M2-1	108	36	3	T -> A	2.70%	183	transversion	
7686	M2-1	144	48	3	G -> A	1.60%	182	transition	
7701	M2-1	159	53	3	C -> T	3.80%	182	transition	
7716	M2-1	174	58	3	A -> T	32.60%	181	transversion	
7771	M2-1	229	77	1	G -> A	1.10%	182	transition	V -> I
7782	M2-1	240	80	3	A -> T	1.10%	182	transversion	
7788	M2-1	246	82	3	T -> C	1.10%	182	transition	
7791	M2-1	249	83	3	T -> C	1.10%	182	transition	
7802	M2-1	260	87	2	T -> C	1.10%	182	transition	I -> T
7842	M2-1	300	100	3	C -> T	3.30%	182	transition	
7851	M2-1	309	103	3	C -> T	80.20%	182	transition	
7866	M2-1	324	108	3	C -> T	1.10%	182	transition	
7891	M2-1	349	117	1	C -> A	88.50%	183	transversion	H -> N
7899	M2-1	357	119	3	A -> G	99.50%	183	transition	
7901	M2-1	359	120	2	C -> T	1.10%	183	transition	P -> L
7902	M2-1	360	120	3	A -> C	1.60%	183	transversion	
7902	M2-1	360	120	3	A -> G	1.10%	183	transition	
7904	M2-1	362	121	2	G -> A	99.50%	183	transition	S -> N
7917	M2-1	375	125	3	A -> G	1.10%	184	transition	
7929	M2-1	387	129	3	T -> C	1.60%	184	transition	
7971	M2-1	429	143	3	A -> G	1.10%	184	transition	
7986	M2-1	444	148	3	G -> A	3.80%	184	transition	
8034	M2-1	492	164	3	A -> G	1.60%	184	transition	
8052	M2-1	510	170	3	C -> T	4.90%	184	transition	
8091	M2-1	549	183	3	T -> C	2.20%	184	transition	
8100	M2-1	558	186	3	C -> T	1.60%	184	transition	
8101	M2-1	559	187	1	C -> T	3.80%	184	transition	H -> Y
8105	M2-1	563	188	2	C -> T	1.10%	184	transition	A -> V
8105	M2-2	4	2	1	CCA -> TCT	1.10%	184	Substitution	P -> S
8107	M2-1	565	189	1	A -> T	1.10%	184	transversion	
8128	M2-2	27	9	3	C -> T	3.80%	184	transition	
8149	M2-2	48	16	3	T -> C	1.10%	182	transition	
8156	M2-2	55	19	1	C -> A	1.10%	182	transversion	L -> I
8166	M2-2	65	22	2	G -> A	2.20%	182	transition	S -> N
8167	M2-2	66	22	3	T -> C	1.10%	182	transition	
8185	M2-2	84	28	3	G -> A	2.70%	182	transition	M -> I
8215	M2-2	114	38	3	T -> C	1.10%	182	transition	
8232	M2-2	131	44	2	A -> G	2.20%	182	transition	N -> S
8246	M2-2	145	49	1	C -> T	2.20%	182	transition	P -> S
8251	M2-2	150	50	3	T -> A	1.10%	183	transversion	D -> E
8263	M2-2	162	54	3	T -> C	3.30%	183	transition	
8280	M2-2	179	60	2	C -> T	2.20%	183	transition	S -> F
8287	M2-2	186	62	3	C -> T	1.10%	182	transition	
8294	M2-2	193	65	1	G -> A	1.60%	182	transition	D -> N
8302	M2-2	201	67	3	T -> C	1.60%	182	transition	
8342	M2-2	241	81	1	T -> C	3.30%	182	transition	Y -> H
8359	M2-2	258	86	3	A -> G	37.90%	182	transition	
8378					C -> T	2.20%	182	transition	
8402					T -> C	1.60%	182	transition	
8421					T -> A	99.50%	182	transversion	
8459	L	25	9	1	T -> A	4.90%	182	transversion	S -> T
8521	L	87	29	3	T -> C	98.40%	182	transition	

8524	L	90	30	3	C -> T	84.60%	182	transition	
8536	L	102	34	3	C -> T	4.90%	183	transition	
8546	L	112	38	1	G -> A	1.60%	183	transition	G -> S
8557	L	123	41	3	C -> T	1.10%	183	transition	
8639	L	205	69	1	T -> A	35.70%	182	transversion	S -> T
8641	L	207	69	3	C -> T	37.40%	182	transition	
8653	L	219	73	3	G -> A	2.20%	183	transition	
8668	L	234	78	3	A -> G	1.60%	183	transition	
8731	L	297	99	3	G -> A	2.20%	182	transition	
8731	L	297	99	3	G -> T	2.70%	182	transversion	
8767	L	333	111	3	G -> A	2.80%	181	transition	
8791	L	357	119	3	C -> T	1.10%	181	transition	
8797	L	363	121	3	T -> C	2.20%	181	transition	
8867	L	433	145	1	C -> A	1.10%	183	transversion	Q -> K
8887	L	453	151	3	T -> C	84.10%	182	transition	
8890	L	456	152	3	T -> C	5.50%	182	transition	
8896	L	462	154	3	T -> C	99.50%	182	transition	
8962	L	528	176	3	T -> C	4.40%	182	transition	
8974	L	540	180	3	A -> C	2.20%	182	transversion	K -> N
8985	L	551	184	2	C -> T	9.30%	182	transition	T -> I
8989	L	555	185	3	C -> T	1.60%	182	transition	
9004	L	570	190	3	G -> A	9.30%	182	transition	
9034	L	600	200	3	T -> A	3.30%	183	transversion	
9073	L	639	213	3	A -> G	2.70%	183	transition	
9088	L	654	218	3	A -> G	1.10%	183	transition	
9094	L	660	220	3	G -> A	88.50%	183	transition	
9097	L	663	221	3	T -> C	1.10%	183	transition	
9133	L	699	233	3	G -> A	1.10%	184	transition	
9208	L	774	258	3	C -> T	2.20%	183	transition	
9247	L	813	271	3	A -> G	2.70%	182	transition	
9274	L	840	280	3	A -> G	6.60%	183	transition	
9277	L	843	281	3	T -> C	1.10%	183	transition	
9313	L	879	293	3	C -> T	6.50%	184	transition	
9364	L	930	310	3	G -> A	1.10%	183	transition	
9377	L	943	315	1	C -> A	1.10%	184	transversion	L -> I
9403	L	969	323	3	A -> G	1.10%	184	transition	
9409	L	975	325	3	C -> T	1.10%	184	transition	
9436	L	1002	334	3	G -> A	2.70%	184	transition	
9437	L	1003	335	1	G -> A	1.10%	184	transition	V -> I
9442	L	1008	336	3	G -> A	4.30%	184	transition	
9457	L	1023	341	3	T -> C	37.20%	183	transition	
9517	L	1083	361	3	C -> T	3.80%	183	transition	
9535	L	1101	367	3	C -> T	2.20%	183	transition	
9538	L	1104	368	3	T -> A	4.40%	183	transversion	
9538	L	1104	368	3	T -> C	83.60%	183	transition	
9541	L	1107	369	3	T -> C	83.60%	183	transition	
9550	L	1116	372	3	G -> A	88.00%	183	transition	
9559	L	1125	375	3	G -> T	1.10%	183	transversion	
9610	L	1176	392	3	A -> C	2.20%	183	transversion	
9644	L	1210	404	1	T -> C	1.10%	184	transition	F -> L
9646	L	1212	404	3	C -> T	3.80%	182	transition	
9649	L	1215	405	3	T -> A	88.00%	184	transversion	
9689	L	1255	419	1	C -> T	1.10%	184	transition	
9715	L	1281	427	3	A -> G	8.20%	184	transition	
9751	L	1317	439	3	C -> T	2.20%	184	transition	
9766	L	1332	444	3	G -> A	98.40%	184	transition	
9796	L	1362	454	3	G -> A	1.10%	184	transition	
9859	L	1425	475	3	T -> C	1.60%	183	transition	
9898	L	1464	488	3	T -> C	84.20%	183	transition	
9933	L	1499	500	2	A -> G	1.10%	183	transition	N -> S
9955	L	1521	507	3	A -> G	4.90%	183	transition	
9991	L	1557	519	3	A -> G	4.90%	183	transition	
10015	L	1581	527	3	G -> A	2.20%	183	transition	
10018	L	1584	528	3	C -> T	99.50%	183	transition	
10033	L	1599	533	3	T -> C	24.00%	183	transition	
10066	L	1632	544	3	T -> A	1.10%	183	transversion	
10111	L	1677	559	3	G -> A	3.30%	183	transition	
10150	L	1716	572	3	A -> G	10.40%	182	transition	
10177	L	1743	581	3	A -> G	1.10%	182	transition	
10207	L	1773	591	3	A -> G	1.10%	182	transition	
10216	L	1782	594	3	A -> G	2.20%	182	transition	
10226	L	1792	598	1	T -> C	73.80%	183	transition	Y -> H
10226	L	1792	598	1	TAC -> CAT	10.40%	183	Substitution	Y -> H
10229	L	1795	599	1	A -> G	1.60%	183	transition	N -> D
10230	L	1796	599	2	A -> C	3.80%	183	transversion	N -> T
10261	L	1827	609	3	T -> C	99.50%	184	transition	
10264	L	1830	610	3	G -> T	1.10%	184	transversion	
10280	L	1846	616	1	T -> C	1.10%	184	transition	
10337	L	1903	635	1	A -> G	1.10%	184	transition	M -> V
10360	L	1926	642	3	A -> G	1.10%	184	transition	
10378	L	1944	648	3	T -> A	88.00%	184	transversion	
10384	L	1950	650	3	C -> T	2.20%	184	transition	
10522	L	2088	696	3	T -> C	1.10%	184	transition	
10585	L	2151	717	3	C -> T	88.50%	182	transition	
10591	L	2157	719	3	A -> G	33.00%	182	transition	
10627	L	2193	731	3	C -> T	1.60%	183	transition	
10639	L	2205	735	3	A -> G	10.40%	183	transition	

10645	L	2211	737	3	T -> A	3.90%	181	transversion	
10700	L	2266	756	1	C -> T	2.20%	182	transition	H -> Y
10705	L	2271	757	3	T -> C	3.30%	182	transition	
10739	L	2305	769	1	T -> C	84.60%	182	transition	
10744	L	2310	770	3	T -> C	1.10%	182	transition	
10804	L	2370	790	3	C -> A	99.40%	180	transversion	
10808	L	2374	792	1	C -> T	1.70%	180	transition	
10810	L	2376	792	3	A -> G	3.90%	180	transition	
10858	L	2424	808	3	T -> C	1.10%	181	transition	
10909	L	2475	825	3	A -> G	1.60%	182	transition	
10912	L	2478	826	3	T -> C	1.10%	182	transition	
10927	L	2493	831	3	A -> G	1.10%	182	transition	
10957	L	2523	841	3	T -> C	89.00%	182	transition	
10966	L	2532	844	3	G -> A	1.60%	182	transition	
10972	L	2538	846	3	A -> G	1.60%	182	transition	
11020	L	2586	862	3	C -> A	88.00%	183	transversion	
11038	L	2604	868	3	T -> C	8.70%	183	transition	
11050	L	2616	872	3	G -> A	9.80%	183	transition	
11062	L	2628	876	3	C -> T	1.10%	183	transition	
11068	L	2634	878	3	A -> G	3.80%	183	transition	
11107	L	2673	891	3	A -> G	1.10%	183	transition	
11143	L	2709	903	3	G -> A	1.10%	181	transition	
11176	L	2742	914	3	G -> A	99.40%	181	transition	
11248	L	2814	938	3	A -> G	1.10%	178	transition	
11261	L	2827	943	1	A -> C	3.40%	178	transversion	N -> H
11296	L	2862	954	3	C -> T	89.30%	178	transition	
11317	L	2883	961	3	C -> T	2.20%	178	transition	
11323	L	2889	963	3	A -> G	1.10%	178	transition	
11375	L	2941	981	1	T -> C	3.90%	181	transition	
11509	L	3075	1025	3	A -> G	1.10%	181	transition	
11521	L	3087	1029	3	A -> G	1.10%	181	transition	
11528	L	3094	1032	1	T -> G	5.00%	181	transversion	S -> A
11560	L	3126	1042	3	C -> T	1.10%	181	transition	
11569	L	3135	1045	3	A -> G	97.80%	181	transition	
11575	L	3141	1047	3	C -> T	38.10%	181	transition	
11581	L	3147	1049	3	C -> T	6.60%	181	transition	
11596	L	3162	1054	3	C -> T	1.70%	181	transition	
11713	L	3279	1093	3	C -> T	89.00%	182	transition	
11752	L	3318	1106	3	C -> T	89.00%	181	transition	
11798	L	3364	1122	1	C -> T	1.10%	181	transition	
11821	L	3387	1129	3	A -> G	1.70%	181	transition	
11854	L	3420	1140	3	T -> C	1.10%	180	transition	
11929	L	3495	1165	3	T -> C	88.50%	182	transition	
11991	L	3557	1186	2	G -> A	1.10%	181	transition	R -> K
12005	L	3571	1191	1	A -> G	1.10%	182	transition	I -> V
12013	L	3579	1193	3	T -> C	1.10%	182	transition	
12025	L	3591	1197	3	A -> G	1.10%	182	transition	
12076	L	3642	1214	3	C -> T	6.60%	182	transition	
12142	L	3708	1236	3	T -> C	1.60%	182	transition	
12256	L	3822	1274	3	A -> T	1.60%	182	transversion	
12350	L	3916	1306	1	G -> A	3.30%	184	transition	D -> N
12364	L	3930	1310	3	G -> A	1.10%	184	transition	
12418	L	3984	1328	3	T -> C	88.00%	184	transition	
12454	L	4020	1340	3	T -> A	3.30%	184	transversion	
12487	L	4053	1351	3	A -> T	1.10%	184	transversion	
12544	L	4110	1370	3	C -> T	1.60%	184	transition	
12556	L	4122	1374	3	A -> G	81.50%	184	transition	
12625	L	4191	1397	3	A -> T	83.20%	184	transversion	
12637	L	4203	1401	3	A -> G	32.60%	184	transition	
12648	L	4214	1405	2	T -> C	1.10%	184	transition	V -> A
12667	L	4233	1411	3	A -> T	87.50%	184	transversion	
12676	L	4242	1414	3	C -> T	3.80%	184	transition	
12679	L	4245	1415	3	G -> A	35.30%	184	transition	
12697	L	4263	1421	3	G -> A	1.10%	184	transition	
12710	L	4276	1426	1	A -> G	1.10%	184	transition	I -> V
12793	L	4359	1453	3	G -> A	2.70%	184	transition	
12799	L	4365	1455	3	A -> G	37.00%	184	transition	
12870	L	4436	1479	2	C -> T	1.10%	184	transition	A -> V
12871	L	4437	1479	3	G -> A	2.20%	184	transition	
12895	L	4461	1487	3	T -> C	1.10%	184	transition	
12898	L	4464	1488	3	T -> C	1.10%	184	transition	
12905	L	4471	1491	1	A -> G	1.10%	184	transition	I -> V
12907	L	4473	1491	3	T -> C	1.10%	184	transition	
12922	L	4488	1496	3	A -> G	1.10%	184	transition	
13033	L	4599	1533	3	T -> C	1.10%	181	transition	
13060	L	4626	1542	3	C -> A	1.60%	183	transversion	
13066	L	4632	1544	3	T -> C	2.20%	183	transition	
13069	L	4635	1545	3	T -> C	8.80%	182	transition	
13072	L	4638	1546	3	T -> C	2.20%	183	transition	
13082	L	4648	1550	1	G -> A	2.20%	183	transition	G -> S
13117	L	4683	1561	3	A -> T	1.10%	184	transversion	
13162	L	4728	1576	3	G -> A	1.10%	182	transition	
13180	L	4746	1582	3	T -> C	7.10%	183	transition	
13210	L	4776	1592	3	A -> C	8.70%	183	transversion	
13210	L	4776	1592	3	A -> T	90.70%	183	transversion	
13213	L	4779	1593	3	C -> T	5.50%	183	transition	
13223	L	4789	1597	1	A -> T	1.10%	183	transversion	S -> C

13315	L	4881	1627	3	T -> A	1.10%	183	transversion	
13344	L	4910	1637	2	A -> G	1.10%	181	transition	K -> R
13348	L	4914	1638	3	A -> T	1.60%	182	transversion	
13369	L	4935	1645	3	A -> T	99.40%	180	transversion	
13435	L	5001	1667	3	A -> G	88.80%	179	transition	
13453	L	5019	1673	3	T -> G	88.80%	179	transversion	
13456	L	5022	1674	3	T -> C	1.10%	179	transition	
13519	L	5085	1695	3	T -> C	2.20%	179	transition	
13522	L	5088	1696	3	T -> C	88.80%	179	transition	
13558	L	5124	1708	3	T -> C	88.90%	180	transition	
13570	L	5136	1712	3	C -> T	2.20%	180	transition	
13579	L	5145	1715	3	C -> T	99.40%	180	transition	
13582	L	5148	1716	3	A -> G	2.20%	180	transition	I -> M
13608	L	5174	1725	2	AT -> GA	6.70%	180	Substitution	D -> G
13609	L	5175	1725	3	T -> A	82.20%	180	transversion	D -> E
13609	L	5175	1725	3	T -> C	4.40%	180	transition	
13611	L	5177	1726	2	A -> G	39.80%	181	transition	K -> R
13615	L	5181	1727	3	G -> A	1.10%	181	transition	
13623	L	5189	1730	2	G -> A	1.10%	180	transition	S -> N
13639	L	5205	1735	3	T -> C	4.90%	182	transition	
13646	L	5212	1738	1	G -> A	3.80%	182	transition	V -> I
13648	L	5214	1738	3	T -> A	2.20%	182	transversion	
13651	L	5217	1739	3	C -> T	1.10%	182	transition	
13664	L	5230	1744	1	C -> T	2.70%	182	transition	P -> S
13674	L	5240	1747	2	C -> T	1.10%	182	transition	S -> F
13681	L	5247	1749	3	G -> A	1.10%	182	transition	
13695	L	5261	1754	2	C -> T	1.60%	182	transition	S -> L
13720	L	5286	1762	3	C -> T	2.70%	182	transition	
13725	L	5291	1764	2	G -> A	1.10%	181	transition	R -> K
13736	L	5302	1768	1	T -> C	1.10%	182	transition	Y -> H
13753	L	5319	1773	3	A -> G	86.80%	182	transition	
13792	L	5358	1786	3	A -> T	5.50%	182	transversion	
13795	L	5361	1787	3	C -> T	1.10%	182	transition	
13837	L	5403	1801	3	C -> A	4.90%	182	transversion	
13891	L	5457	1819	3	T -> C	2.20%	182	transition	
13906	L	5472	1824	3	A -> G	1.10%	181	transition	
13960	L	5526	1842	3	A -> G	35.90%	181	transition	
13969	L	5535	1845	3	T -> A	35.90%	181	transversion	D -> E
13981	L	5547	1849	3	A -> G	1.10%	181	transition	I -> M
13993	L	5559	1853	3	T -> C	3.80%	183	transition	
14122	L	5688	1896	3	G -> A	88.00%	184	transition	
14179	L	5745	1915	3	C -> T	1.60%	183	transition	
14251	L	5817	1939	3	G -> A	3.80%	183	transition	
14261	L	5827	1943	1	G -> A	2.20%	183	transition	V -> I
14302	L	5868	1956	3	A -> G	1.10%	184	transition	
14311	L	5877	1959	3	C -> T	37.50%	184	transition	
14374	L	5940	1980	3	C -> T	1.10%	184	transition	
14425	L	5991	1997	3	C -> T	2.20%	184	transition	
14491	L	6057	2019	3	A -> G	4.30%	184	transition	
14506	L	6072	2024	3	A -> G	1.10%	183	transition	
14522	L	6088	2030	1	C -> T	1.10%	183	transition	
14614	L	6180	2060	3	C -> T	1.10%	183	transition	
14629	L	6195	2065	3	A -> G	2.20%	183	transition	
14656	L	6222	2074	3	T -> A	99.50%	182	transversion	F -> L
14665	L	6231	2077	3	C -> T	99.50%	182	transition	
14669	L	6235	2079	1	A -> G	1.10%	182	transition	S -> G
14675	L	6241	2081	1	G -> A	1.10%	182	transition	D -> N
14686	L	6252	2084	3	G -> A	89.30%	182	transition	
14757	L	6323	2108	2	A -> G	1.10%	182	transition	K -> R
14797	L	6363	2121	3	A -> T	3.90%	181	transversion	L -> F
14803	L	6369	2123	3	T -> C	3.90%	181	transition	
14806	L	6372	2124	3	T -> C	3.90%	181	transition	
14819	L	6385	2129	1	G -> A	1.10%	180	transition	V -> I
14884	L	6450	2150	3	A -> G	1.10%	179	transition	
