# Supplementary material for: Whole genome analysis of local Kenyan and global sequences unravels the epidemiological and molecular evolutionary dynamics of RSV genotype ON1 strains
Source: Virus Evol. 2018 Sep 24;4(2):vey027. doi: 10.1093/ve/vey027 (PMC6153471; doi:10.1093/ve/vey027)
Supplement: Supplementary S4 Table [file vey027_supplementary_s4_table.pdf]

CDS	CDS Nt Pos.	SNP Codon Pos.	CDS AA Pos.	Change	AA Change	SNP Type
NS2	66	3	22	G -> A		Transition
NS2	88	1	30	C -> T		Transition
N	531	3	177	A -> T		Transversion
N	1107	3	369	T -> C		Transition
P	477	3	159	C -> T		Transition
P	546	3	182	A -> G		Transition
M	18	3	6	T -> C		Transition
M	198	3	66	G -> A		Transition
M	204	3	68	C -> T		Transition
M	504	3	168	C -> T		Transition
G	69	3	23	G -> A		Transition
G	424	1	142	TT -> CA	L -> Q	Substitution
G	622	1	208	C -> A	L -> I	Transversion
G	681	3	227	T -> C		Transition
G	690	3	230	T -> C		Transition
G	695	2	232	G -> A	G -> E	Transition
G	709	1	237	A -> G	N -> D	Transition
G	717	3	239	T -> C		Transition
G	758	2	253	A -> C	K -> T	Transversion
G	817	1	273	T -> A	Y -> N	Transversion
G	821	2	274	C -> T	P -> L	Transition
G	840	3	280	C -> T		Transition
G	851	2	284	-GTCAAGAGGAAACCCTCCACTCAACCACCCCCGAAGGCCATCCAAGCCCATCACAAGTCCATACAACATCCG	GQEETLHSTTSEGYPSPSQVHTTSE -> E	Deletion
G	929	2	310	C -> T	P -> L	Transition
G	941	2	314	T -> C	L -> P	Transition
F	346	1	116	A -> G	N -> D	Transition
F	364	1	122	G -> A	A -> T	Transition
F	390	3	130	T -> C		Transition
F	573	3	191	G -> A		Transition
F	1095	3	365	G -> A		Transition
F	1146	3	382	C -> T		Transition
F	1587	3	529	C -> T		Transition
F	1629	3	543	A -> T		Transversion
F	1632	3	544	T -> A		Transversion
M2-1	309	3	103	T -> C		Transition
M2-1	349	1	117	A -> C	N -> H	Transversion
L	90	3	30	T -> C		Transition
L	453	3	151	C -> T		Transition
L	660	3	220	A -> G		Transition
L	1104	3	368	C -> T		Transition
L	1107	3	369	C -> T		Transition
L	1116	3	372	A -> G		Transition
L	1215	3	405	A -> T		Transversion
L	1464	3	488	C -> T		Transition
L	1792	1	598	C -> T	H -> Y	Transition
L	1944	3	648	A -> T		Transversion
L	2151	3	717	T -> C		Transition
L	2305	1	769	C -> T		Transition
L	2523	3	841	C -> T		Transition
L	2586	3	862	A -> C		Transversion
L	2862	3	954	T -> C		Transition
L	3279	3	1093	T -> C		Transition
L	3318	3	1106	T -> C		Transition
L	3495	3	1165	C -> T		Transition
L	3984	3	1328	C -> T		Transition
L	4122	3	1374	G -> A		Transition
L	4191	3	1397	T -> A		Transversion
L	4233	3	1411	T -> A		Transversion
L	5001	3	1667	G -> A		Transition
L	5019	3	1673	G -> T		Transversion

L	5088	3	1696	C -> T		Transition
L	5124	3	1708	C -> T		Transition
L	5175	3	1725	A -> T	E -> D	Transversion
L	5319	3	1773	G -> A		Transition
L	5688	3	1896	A -> G		Transition
L	6252	3	2084	A -> G		Transition
