# Supplementary material for: Whole genome analysis of local Kenyan and global sequences unravels the epidemiological and molecular evolutionary dynamics of RSV genotype ON1 strains
Source: Virus Evol. 2018 Sep 24;4(2):vey027. doi: 10.1093/ve/vey027 (PMC6153471; doi:10.1093/ve/vey027)
Supplement: Supplementary S5 Table [file vey027_supplementary_s5_table.pdf]

CDS	CDS Nt Pos.	SNP Codon Pos.	CDS AA Pos.	Change	AA Change	SNP Type
N	151	1	51	T -> C		Transition
N	558	3	186	T -> C		Transition
N	561	3	187	T -> C		Transition
N	717	3	239	T -> C		Transition
P	567	3	189	T -> C		Transition
M	111	3	37	C -> T		Transition
M	123	3	41	A -> G		Transition
G	69	3	23	G -> A		Transition
G	383	2	128	C -> T	S -> F	Transition
G	821	2	274	C -> T	P -> L	Transition
G	840	3	280	C -> T		Transition
G	893	2	298	C -> T	P -> L	Transition
G	910	1	304	C -> T	H -> Y	Transition
G	929	2	310	C -> T	P -> L	Transition
F	309	3	103	C -> T		Transition
F	364	1	122	G -> A	A -> T	Transition
F	516	3	172	A -> G		Transition
F	1507	1	503	C -> A	L -> I	Transversion
M2-1	549	3	183	T -> C		Transition
M2-2	131	2	44	A -> G	N -> S	Transition
L	219	3	73	G -> A		Transition
L	297	3	99	G -> T		Transversion
L	528	3	176	T -> C		Transition
L	540	3	180	A -> C	K -> N	Transversion
L	774	3	258	C -> T		Transition
L	1002	3	334	G -> A		Transition
L	1101	3	367	C -> T		Transition
L	1599	3	533	T -> C		Transition
L	1782	3	594	A -> G		Transition
L	4122	3	1374	G -> A		Transition
L	4437	3	1479	G -> A		Transition
L	5457	3	1819	T -> C		Transition
L	5991	3	1997	C -> T		Transition
