# Supplementary material for: Whole genome analysis of local Kenyan and global sequences unravels the epidemiological and molecular evolutionary dynamics of RSV genotype ON1 strains
Source: Virus Evol. 2018 Sep 24;4(2):vey027. doi: 10.1093/ve/vey027 (PMC6153471; doi:10.1093/ve/vey027)
Supplement: Supplementary S6 Table [file vey027_supplementary_s6_table.pdf]

GENE	SELECTION TEST/ANALYSIS METHOD						
	FUBAR	SLAC	FEL	MEME	Positive selection by all methods	BUSTED	aBSREL
NS1	22**, 23**, 44**, 48**, 67**, 71**, 76**, 94**, 139**	-	22**, 23**, 44**, 71**, 76**, 94**	-	-	Yes	None
NS2	15*, 22**, 70**	22**	19**, 20**, 22**, 54**, 66**, 72**, 96**	-	-	None	None
N	30**, 35**, 49**, 51**, 86**, 100**, 137**, 147**, 153**, 170**, 172**, 177**, 185**, 186**, 205**, 243**, 247**, 253**, 259**, 264**, 267**, 268**, 279**, 316**, 326**, 335**, 350**, 351**, 372**	51**, 153**, 186**, 267**, 259**	30**, 45**, 49**, 51**, 86**, 100**, 137**, 147**, 153**, 170**, 172**, 177**, 185**, 186**, 187**, 205**, 243**, 247**, 253**, 259**, 264**, 267**, 268**, 279**, 326**, 335**, 345**, 350**, 351**, 360**, 372**, 386**	-	-	None	None
P	49**, 68**, 69**, 77**, 98**, 99**, 103**, 129**, 155**, 157**, 163**, 171**, 182**, 184**, 225**	98**, 103**, 129**, 155**, 171**, 225**	39**, 49**, 68**, 69**, 77**, 98**, 99**, 103**, 129**, 155**, 157**, 163**, 171**, 182**, 184**, 194**, 212**, 225**, 240**	-	-	None	None
M	6**, 37**, 41**, 51**, 82**, 92**, 122**, 148**, 157**, 165**, 174**, 188**, 196**, 198**, 208**, 210**, 218**, 250**	6**, 196**, 208**	6**, 37**, 41**, 51**, 82**, 92**, 122**, 148**, 157**, 165**, 188**, 196**, 198**, 208**, 210**, 218**, 250**	-	-	Yes	None
SH	42**	42**	19**, 32**, 42**	-	-	None	None
G	20**, 23**, 80**, 143**, 201*, 202**, 227**, 228**, 239**, 250*, 251*, 310*, 319**	80**, 143**, 227**, 239**, 284*, 310*, 319**	14**, 20**, 23**, 80**, 143**, 176**, 186**, 201*, 202**, 226**, 227**, 228**, 230**, 239**, 251*, 265**, 273*, 289**, 297*, 310*, 319**	73*, 201*, 251*, 273*, 310*	310	None	None
F	6**, 12**, 22**, 26**, 60**, 73**, 100**, 110**, 111**, 112**, 148**, 149**, 154**, 159**, 172**, 182**, 191**, 196**, 215**, 223**, 226**, 259**, 293**, 308**, 348**, 360**, 365**, 405**, 411**, 415**, 422**, 457**, 504**, 528**, 534**, 543**, 555**, 560**, 568**	22**, 73**, 159**, 223**, 405**, 422**, 457**, 534**, 560**, 568**	6**, 22**, 26**, 60**, 73**, 100**, 110**, 112**, 148**, 149**, 154**, 159**, 172**, 182**, 196**, 215**, 223**, 226**, 259**, 293**, 308**, 348**, 360**, 405**, 411**, 415**, 422**, 457**, 504**, 528**, 534**, 543**, 555**, 560**, 568**	-	-	None	None
M2-1	80**, 103**, 120**, 143**, 150**, 186**	103**, 143**, 186**	103**, 125**, 143**, 150**, 164**, 186**	-	-	None	None
M2-2	2**	-	2**, 8**	-	-	None	None
L	22**, 28**, 29**, 30**, 34**, 43**, 73**, 78**, 82**, 99**, 102**, 111**, 114**, 119**, 121**, 152**, 153**, 154**, 176**, 185**, 200**, 210**, 212**, 213**, 218**, 220**, 221**, 258**, 271**, 276**, 280**, 281**, 293**, 296**, 314**, 324**, 325**, 334**, 336**, 341**, 349**, 361**, 367**, 368**, 369**, 372**, 375**, 392**, 404**, 405**, 413**, 427**, 436**, 439**, 444**, 446**, 448**, 462**, 463**, 475**, 487**, 488**, 507**, 518**, 526**, 528**, 533**, 540**, 544**, 559**, 563**, 572**, 581**, 591**, 594**, 609**, 610**, 613**, 617**, 618**, 639**, 642**, 648**, 650**, 662**, 689**, 696**, 717**, 719**, 731**, 735**, 737**, 749**, 757**, 758**, 760**, 769**, 770**, 777**, 778**, 790**, 792**, 798**, 808**, 825**, 826**, 831**, 837**, 841**, 846**, 862**, 863**, 868**, 872**, 876**, 878**, 883**, 891**, 892**, 903**, 914**, 933**, 938**, 954**, 961**, 963**, 1004**, 1006**, 1007**, 1016**, 1025**, 1029**, 1033**, 1034**, 1035**, 1042**, 1045**, 1047**, 1049**, 1050**, 1054**, 1074**, 1082**, 1093**, 1094**, 1106**, 1129**, 1135**, 1140**, 1144**, 1145**, 1178**, 1193**, 1197**, 1199**, 1214**, 1236**, 1274**, 1282**, 1286**, 1304**, 1305**, 1310**, 1328**, 1340**, 1345**, 1351**, 1358**, 1362**, 1370**, 1374**, 1376**, 1381**, 1392**, 1397**, 1401**, 1402**, 1408**, 1409**, 1411**, 1414**, 1415**, 1427**, 1435**, 1436**, 1455**, 1483**, 1487**, 1488**, 1496**, 1510**, 1513**, 1522**, 1533**, 1534**, 1542**, 1544**, 1545**, 1546**, 1554**, 1555**, 1561**, 1566**, 1569**, 1576**, 1582**, 1592**, 1593**, 1606**, 1627**, 1638**, 1645**, 1647**, 1667**, 1674**, 1679**, 1681**, 1684**, 1695**, 1696**, 1700**, 1708**, 1712**, 1715**, 1728**, 1735**, 1739**, 1749**, 1755**, 1762**, 1771**, 1773**, 1786**, 1787**, 1799**, 1801**, 1813**, 1819**, 1824**, 1853**, 1868**, 1894**, 1896**, 1900**, 1909**, 1911**, 1915**, 1927**, 1931**, 1944**, 1956**, 1959**, 1961**, 1980**, 2019**, 2024**, 2033**, 2042**, 2060**, 2061**, 2065**, 2075**, 2077**, 2084**, 2095**, 2107**, 2123**, 2124**, 2135**, 2150**	29**, 99**, 121**, 176**, 287**, 392**, 444**, 475**, 533**, 790**, 876**, 954**, 1054**, 1214**, 1297**, 1411**, 1653**, 1773**	22**, 28**, 29**, 30**, 34**, 43**, 78**, 82**, 99**, 114**, 119**, 121**, 153**, 176**, 200**, 212**, 213**, 221**, 271**, 281**, 334**, 336**, 349**, 368**, 392**, 427**, 436**, 439**, 444**, 446**, 462**, 463**, 475**, 507**, 518**, 528**, 533**, 544**, 572**, 581**, 591**, 594**, 617**, 639**, 648**, 662**, 717**, 737**, 749**, 758**, 760**, 770**, 778**, 790**, 808**, 825**, 826**, 837**, 846**, 862**, 868**, 872**, 876**, 878**, 883**, 891**, 914**, 938**, 954**, 963**, 1006**, 1016**, 1025**, 1033**, 1034**, 1035**, 1045**, 1050**, 1054**, 1106**, 1140**, 1144**, 1145**, 1193**, 1199**, 1214**, 1236**, 1274**, 1286**, 1340**, 1345**, 1351**, 1358**, 1376**, 1381**, 1397**, 1402**, 1409**, 1411**, 1455**, 1483**, 1487**, 1488**, 1513**, 1522**, 1533**, 1544**, 1545**, 1546**, 1561**, 1566**, 1582**, 1592**, 1627**, 1638**, 1647**, 1674**, 1684**, 1695**, 1700**, 1715**, 1728**, 1735**, 1771**, 1773**, 1786**, 1787**, 1813**, 1819**, 1824**, 1853**, 1868**, 1896**, 1900**, 1909**, 1911**, 1927**, 1931**, 1956**, 2019**, 2042**, 2061**, 2065**, 2077**, 2084**, 2123**, 2124**, 2135**, 2150**	2030*, 2122*	-	None	None
KEY:							
*	Positive selection						
**	Negative Selection						
-	No site						
