# Supplementary material for: Whole genome analysis of local Kenyan and global sequences unravels the epidemiological and molecular evolutionary dynamics of RSV genotype ON1 strains
Source: Virus Evol. 2018 Sep 24;4(2):vey027. doi: 10.1093/ve/vey027 (PMC6153471; doi:10.1093/ve/vey027)
Supplement: Supporting Information Legends [file vey027_supporting_information_legends.docx]

**Supporting Information Legends**

**S1 Fig: Sampling locations of the global ON1 G-gene dataset**

A map showing source country locations of the global ON1 G-gene sequences dataset analyzed here with circles representative of relative proportion of contributing sequences by country

**S2 Fig: Samples Ct value distributions**

Histograms showing distribution of Ct values for samples from the (A)

KHDSS and (B) KCH.

**S3 Fig: Root-to-tip regression analysis of Kilifi RSV-A ORFs**

A root-to-tip regression analysis of ML trees from whole genomes and 11 separate coding regions, with the points colour coded by genotype; GA2 (red) and ON1 (cyan).

**S4 Fig: BEAST MCC ON1-GA2 divergence trees for different ORFs**

MCC trees inferred from different ORFs of 184 RSV-A complete genome sequences from Kilifi with the tips colour coded by genotype, i.e. ON1 (cyan) and GA2 (red).

**S1 Table: Model selection to infer time-structured phylogenies**

Log-marginal likelihood estimates using the path-sampling (PS) and stepping-stone (SS) model selection approaches (Drummond *et al.* 2012). The best-fitting clock and population model combination is underscored.

**S2 Table: Study samples and genomes details**

**S3 Table: SNPs identified from dataset of all Kilifi genomes**

**S4 Table: Signature SNPs between ON1 and GA2 viruses**

**S5 Table: Signature SNPs between successful and limited transmission ON1 viruses**

**S6 Table: Sites under selective pressure**
